# Potential anticancer effects of *Peganum harmala* in human papillomavirus-related cervical and head and neck cancer cells

**DOI:** 10.3389/fphar.2025.1668827

**Published:** 2025-11-17

**Authors:** Hiba F. Muddather, Tohfa Nasibova, Gábor J. Szebeni, Nikolett Gémes, Noémi Bózsity, Renáta Minorics, Eldar Garayev, Ilkay Erdogan Orhan, Gaëtan Herbette, Zsuzsanna Schelz, Judit Hohmann, István Zupkó

**Affiliations:** 1 Institute of Pharmacodynamics and Biopharmacy, University of Szeged, Szeged, Hungary; 2 Institute of Pharmacognosy, University of Szeged, Szeged, Hungary; 3 Department of General and Toxicological Chemistry, Azerbaijan Medical University, Baku, Azerbaijan; 4 Laboratory of Functional Genomics, Core Facility, Biological Research Centre, Hungarian Academy of Sciences, Szeged, Hungary; 5 Department of Internal Medicine, Hematology Centre, University of Szeged, Szeged, Hungary; 6 Department of Pharmacognosy, Lokman Hekim University, Ankara, Türkiye; 7 Institut de Chimie des Substances Naturelles (ICSN), Centre National de la Recherche Scientifique UPR 2301, Université Paris Saclay, Gif-sur-Yvette, France; 8 Spectropole, Fédération des Sciences Chimiques de Marseille, Centrale Marseille, CNRS, Aix-Marseille Université, Marseille, France; 9 HUN-REN–USZ Biologically Active Natural Products Research Group, University of Szeged, Szeged, Hungary; 10 Interdisciplinary Centre of Natural Products, University of Szeged, Szeged, Hungary

**Keywords:** *Peganum harmala*, harmine, anticancer, antimetastatic, head and neck cancer, cervical cancer

## Abstract

**Background:**

*P. harmala* L (*P. harmala*) has a longstanding role in ethnomedical treatments. Its reported anticancer effects have led to increased research interest; however, its antineoplastic properties on cervical and head and neck (HN) cancer cells need further investigation. In this study, we investigated *P. harmala’s* antineoplastic effects on HPV-infected cervical and HN cancer cell lines.

**Methods:**

The crude extract derived from multiple plant parts and isolated β-carboline alkaloids was tested on several human neoplastic cell types using the MTT-based analysis. Apoptosis was examined by fluorescent double staining, Annexin V-Alexa 488–propidium iodide labeling, and caspase-3 assays. Moreover, flow cytometry was employed to explore the cell cycle progression alterations, while the tubulin polymerization assay assessed influences on microtubule dynamics. The antimetastatic property was investigated by wound healing and transwell invasion assays to explore the impact on cellular motility and invasiveness, respectively.

**Results:**

The IC_50_ values were calculated and examined relative to non-malignant fibroblasts to assess selective toxicity. The root extract demonstrated the most substantial growth-inhibitory effect among the tested extracts. Harmine, one of the isolated bioactive alkaloids, showed a substantial effect, with inhibitory concentrations between 6.05 and 27.85 µM. Apoptosis induced by harmine was confirmed through cellular morphological appearance, flow cytometric evaluations, and caspase-3 activation. Assessment of the cell cycle demonstrated that harmine disrupted cell cycle progression, particularly increasing the apoptotic sub-G1 and G2/M phase populations. Moreover, it revealed the ability to stabilize microtubules. Our findings showed that harmine and the root extract significantly reduced cell migration. Furthermore, harmine was found to have anti-invasive properties.

**Conclusion:**

These findings showed potential harmine antiproliferative and antimetastatic activities, indicating its potential for further research in developing natural therapeutic agents.

## 1 Introduction

Cancer remains a major contributor to global mortality rates. Approximately 19.3 million new cancer diagnoses occurred, and almost 10 million fatalities were estimated globally (Sung et al., 2021). GLOBOCAN 2020 data revealed that cervical cancer is the fourth most frequent malignancy in women globally, accounting for an estimated 604,127 new cases and 341,831 fatalities (Sung et al., 2021; Singh et al., 2022). In comparison, head and neck (HN) cancer is a crucial type, responsible for 377,713 and 98,412 cases and 177,757 and 48,143 reported deaths for the lip/oral cavity and oropharynx, respectively (Sung et al., 2021).

Cancer presents as a diverse disease with complex risk factors (Wu et al., 2018). The family of human papillomaviruses (HPVs) contains over 170 distinct types, which most commonly infect the genital mucosa, upper respiratory system, and skin. Evidence indicates that oncogenic HPVs play a crucial role in the pathogenesis of cervical cancers and certain types of HN squamous cell carcinoma (Gillison et al., 2014; Tommasino, 2014; Ghittoni et al., 2015; Lu et al., 2022). In recent decades, the global rate of cervical cancer cases and related deaths has declined in many regions (Sung et al., 2021; Singh et al., 2022). In contrast, incidence continued to increase for oral and oropharyngeal cancer (Näsman et al., 2009; Angela Hong et al., 2016; Siegel et al., 2022).

Metastasis is multifaceted and involves multiple sequential steps that ultimately lead to cancer’s outgrowth in a different organ from which it originated (Steeg, 2006; Polacheck et al., 2013; Guan, 2015). Metastatic spread, not the original neoplasm, is responsible for most cancer fatalities (Guan, 2015). Solid tumors at an early stage are often curable, as a result of strides in both early diagnosis and therapeutic modalities (Wells et al., 2013). Therefore, to improve survival rates for most cancer patients, there has been an increasing need to investigate alternative anticancer agents with antimetastatic and anti-invasive properties.

Over the past decades, for their notable anticancer properties, natural products have gained substantial research interest (Harvey, 2008; Newman and Cragg, 2020). *Peganum harmala* L. (Nitrariaceae), often referred to as Espand, harmel, or Syrian rue, is a perennial plant widely growing across North Africa, Central Asia, and the Middle East. Different parts of the *P. harmala* have traditionally been used to manage conditions such as diabetes mellitus, hypertension, asthma, and cancer, and as an antispasmodic, emmenagogue, abortifacient agent, and analgesic (Lamchouri et al., 2000; Pieroni et al., 2005; Farouk et al., 2007; Tahraoui et al., 2007; Frison et al., 2008). The phytochemical profile of *P. harmala* includes a variety of alkaloids; these include β-carbolines and quinazoline derivatives. Harmine, recognized as the key bioactive constituent of *P. harmala*, demonstrates diverse therapeutic properties such as antiplasmodial, antiviral, antimutagenic, antiplatelet, cytotoxic, hallucinogenic effects, and inhibiting monoamine oxidase action (Burger and Xara, 1965; Glennon et al., 2000; Cao et al., 2005; Astulla et al., 2008; Im et al., 2009). For instance, *P. harmala* has demonstrated antiviral activity against a variety of clinically relevant viruses, including herpes simplex virus, influenza viruses, and enterovirus 71 (EV-71), mainly due to its rich content of β-carboline alkaloids that interfere with viral replication and modulate cellular pathways (Chen et al., 2015; 2018; Hegazy et al., 2023). In addition to these antiviral properties, a hydroalcoholic extract of *P. harmala* demonstrated concentration-dependent growth inhibition activity against HPV-positive HeLa cells without additional mechanistic elucidation (Mashhadi et al., 2017). Furthermore, two alkaloids of the plant harmine and harmaline have been investigated in an earlier study using human cancer cell lines, including cervical cells HeLa and C33A. While harmaline demonstrated a general inhibition of cancer cell viability, harmine was active only against C33A cells and decreased their colony-formation capacity (Jiménez et al., 2008). Notably, the antiviral potential of the β-carboline alkaloids has also been extended to the HPV; a prior study revealed that substituted tetrahydro-β-carbolines significantly inhibited HPV replication *in vitro* with low cytotoxicity (Miller et al., 2010). These findings further reinforce the potential that the β-carboline constituents of *P. harmala* may serve as promising candidates for targeting HPV infection and related malignancies.

Extensive *in vitro* and *in vivo* investigations have shown the profound anticancer properties of *P. harmala* (Ayoob et al., 2017; Bournine et al., 2017; Ruan et al., 2017; Mehreen Sadaf et al., 2021). Particularly, harmine was previously examined for its potential antitumor activity, demonstrating its remarkable capacity to modulate diverse cellular processes, including pathways related to the regulation of cell cycle, apoptosis, and metastasis (Hamsa and Kuttan, 2011b; Zhao and Wink, 2013; Sun et al., 2015; Hashemi Sheikh Shabani et al., 2015; Gao et al., 2017; Oodi et al., 2017; Ruan et al., 2017; Ding et al., 2019; Hai-Rong et al., 2019; Nafie et al., 2021; Hu et al., 2024; Akabli et al., 2025). Given that these pathways are commonly dysregulated across numerous malignancies, including HPV-related cancer, harmine’s multifunctional properties may offer a promising therapeutic potential against HPV-related malignancies. Nevertheless, the antimetastatic effect of harmine on cervical and HN cancer cells has yet to be unraveled.

Our current study focused on investigating the antineoplastic effects of extracts obtained from various anatomical sections of *P. harmala* and its isolated β-carboline alkaloids on selected gynecological, breast, and oral squamous cell carcinoma cell lines. Harmine was identified as the most active β-carboline alkaloid. Therefore, in the current study, we investigated harmine’s antiproliferative effects, its effects on cellular morphology, cell cycle distribution, apoptosis induction, and its impact on tubulin polymerization. In addition, the antimigratory and anti-invasive characteristics of harmine have been examined.

## 2 Materials and methods

### 2.1 General experimental procedures

Solvents for extraction and TLC were purchased from Molar Chemicals Kft. (Halásztelek, Hungary). HPLC-grade solvents were acquired from VWR Chemicals International S. A. S. (Fontenay-sous-Bois, France), and HPLC-grade water was purified by a Millipore Direct-QVR 3 UV pump (Millipore S. A. S., Molsheim, France). An ultrasonic bath (VWR Chemicals International S. A. S., Fontenay-sous-Bois, France) was used to dissolve the extracts. NMR spectra were recorded in CDCl_3_ and DMSO-d6 on a Bruker Avance DRX 500 spectrometer at 500 MHz (^1^H) and 125 MHz (^13^C) and a Bruker AM-600 (Avance III, TXI probe) spectrometer at 600 MHz (^1^H) and 150 MHz (^13^C).

### 2.2 Plant material

*P. harmala* plant material was cultivated in July 2020 in the Yevlakh region, Khaldan, Azerbaijan. The whole plants were air-dried, and the plant’s organs were separated into seeds, capsules, stems, flowers, and roots. A voucher specimen No. 943 is stored at the Institute of Pharmacognosy, University of Szeged.

### 2.3 Preparation of the extracts

250 g each of dried and ground plant parts (seeds, capsules, stems, flowers, and roots) were extracted three times with 1 L 95% EtOH. The EtOH extracts were combined and evaporated. These dried residues were extracted with distilled water using a magnetic stirrer to separate the alkaloids from the resins, and then the filtered aqueous extracts were evaporated at 40 °C. At the end of this extraction process, 5.1 g, 7.4 g, 6.1 g, 3.5 g, and 2.7 g of extracts were obtained from the root, seed, stem, capsule, and flower with 2.04%, 2.96%, 2.56%, 1.40%, and 1.08% yields, respectively.

### 2.4 Sources of alkaloids

The alkaloids harmine, harmaline, and harmol were yielded from roots, tetrahydroharmine from seeds, and harmalol, tryptoline, and 5-hydroxytryptophan from stems of *P. harmala*, as described below. The plant was collected in September 2021 in the Zig region of Baku, Azerbaijan. 1 kg of dried, powdered root was exhaustively extracted with 95% EtOH (3 × 3 L) using percolation at room temperature. The hydroalcoholic extract was evaporated, yielding 42.3 g residue. This extract was dissolved in 1 L of 2% aqueous HCl solution and fractionated through a solvent–solvent partition using CHCl_3_ (3 × 1 L). The aqueous-acidic part (1 L) was adjusted to pH nine using NaOH and extracted with chloroform (3 × 1 L). The CHCl_3_ fractions were combined and washed with MeOH. Harmine (346.8 mg) crystallized from the MeOH solution as a white powder. The mother liquor was purified using silica gel-based preparative thin-layer chromatography (PTLC) CHCl_3_–MeOH (8:2) as a developing system, resulting in harmol (1.96 mg) and harmaline (1.03 mg) (Supplementary Figure S1).

Dried and ground *P. harmala* seeds (250 g) were extracted with 3 × 500 mL 95% ethanol. The extract was evaporated to dry, and 200 mL of 5% hydrochloric acid solution was added. The aqueous-acidic solution was extracted with 3 × 200 mL CHCl_3_. Then, the aqueous acid solution was alkalized with 25% NH_3_ to a pH of 9, and this solution was extracted again with CHCl_3_ (4 × 200 mL). The alkaloid-containing CHCl_3_ phase was separated by PTLC on silica gel (SIL G-100 UV254) using a CHCl_3_–MeOH (17:3) as a developing system, yielding tetrahydroharmine, besides harmine and harmaline (Supplementary Figure S2).

For alkaloid isolation, 1 kg of dried, chopped stems was extracted with 95% EtOH (5 × 3 L) at 40 °C for 8 h. Following solvent evaporation, the resulting residue was washed with 4 × 500 mL *n*-hexane, and then the remaining mass was further processed through open column chromatography (OCC) on silica gel 60 (0.040–0.063 mm) (Merck KGaA, Darmstadt, Germany) using mixtures of CHCl_3_ and MeOH (95:5, 90:10, 85:15, 80:20, 75:25, 70:30, 65:35, 60:40, 55:45, and 50:50) as mobile phase, yielding 8 fractions. Fraction 5 was further separated by OCC using the same method, affording 9 subfractions (Fr 5/1–9). Fraction 5/2 contained an alkaloid with turquoise spots in UV_366nm_ light; this compound was identified as harmalol. Fraction 6 was purified through silica gel-based PTLC using a CHCl_3_–MeOH mixture (8:2) as a developing system, and tryptoline (tetrahydronorharmane) (R_f_ = 0.27) was isolated in pure form by this means. The same PTLC method was applied to isolate 5-hydroxytryptophan from fractions 7 (Supplementary Figure S3). The isolated compounds were identified employing 1D and 2D NMR spectroscopy (Supplementary Figures S4–S9). The ^1^H and ^13^C chemical shift assignments were consistent with previously reported findings (Abourashed et al., 2003; Kusurkar and Goswami, 2004; Schieferstein et al., 2015).

### 2.5 Quantitative analysis of alkaloids

The amounts of the main alkaloids, harmine, harmol, and harmaline in the aqueous extracts of various plant organs were quantified by the RP-HPLC method using Phenomenex Luna C18 (2) (5 μm, 100 Å, 150 × 4.6 mm) column (Phenomenex, Torrance, CA, United States). The mobile phase was a gradient system of 25%–55% solvent B in A, which proceeded for 20 min at a flow rate of 1 mL/min. Solvent A consisted of a 2.20% sodium acetate solution, pH-adjusted to 4.0 using acetic acid, and solvent B was MeOH. After every run, a 5-min wash with MeOH and a 10-min re-equilibration period were implemented. Calibration curves were determined using stock solutions of 0.1006 mg/mL harmine, 0.1015 mg/mL harmol, and 0.1006 mg/mL harmaline. The injected volumes were 2, 5, 10, 20, 30, 50 μL. Detection was in UV light at 254 nm and 340 nm. The dried extracts were analyzed after dissolving them in MeOH. Ultrasonication was applied to dissolve 200 mg of each extract in 5 mL of MeOH. After filtration, the solution was diluted in MeOH at a 1:10 ratio. The amount of each alkaloid in the extracts was calculated based on the calibration curves (Supplementary Figures S10, S11). Each measurement was made in triplicate.

### 2.6 Chemicals and cell cultures

Cell lines were primarily purchased from the European Collection of Authenticated Cell Cultures (ECACC, Salisbury, UK), except for C33A and SiHa cell lines obtained from the American Tissue Culture Collection (ATCC, Manassas, VA, United States) and the oral squamous cell carcinoma cell lines acquired from DSMZ–German Collection of Microorganisms and Cell Cultures GmbH (Braunschweig, Germany). Cells were cultured in Eagle’s Minimum Essential Medium (EMEM) supplied with 1% solution of non-essential amino acids mixture, 10% heat-inactivated fetal bovine serum (FBS), and 1% antibiotic-antimycotic solution containing penicillin/streptomycin and amphotericin B. For culturing the UPCI-SCC-154 cell line, additional supplementation of 1% L-glutamine has been done to the media. The cells were incubated under a humidified 5% CO_2_ atmosphere at 37 °C, and used for experiments up to passage 30. Cell lines were regularly tested for *mycoplasma* using the MycoAlert PLUS Detection Kit (Lonza, Basel, Switzerland). Unless otherwise indicated, media and supplements used for culturing the cells and experimental procedures were obtained from Capricorn Scientific GmbH (Ebsdorfergrund, Germany). Chemicals and assay kits used throughout the experiments were acquired from Merck Life Science Ltd. (Budapest, Hungary).

### 2.7 MTT antiproliferative assay

*P. harmala’s* growth inhibitory activities and tumor selectivity were assessed across a panel of gynecological, breast, and oral squamous cell carcinoma cell lines via a standard MTT assay protocol (Mosmann, 1983). NIH/3T3 non-neoplastic murine fibroblast cells were utilized to identify cancer selectivity. Briefly, cells were plated into 96-well plates at a density of 5000 cells per well, with the exception of C33A cells, which were seeded at a higher cell density of 10,000 cells per well and allowed to adhere overnight in the incubator. Subsequently, cells were exposed to the medium supplemented with the desired concentrations of the tested substances and incubated for a further 72 h. Afterward, 20 µL of MTT reagent (5 mg/mL) solution was dispensed into each well, followed by an additional 4 h incubation. To solubilize the formazan precipitate, 100 µL DMSO was introduced into each well. Absorbances were measured at 545 nm using a microplate spectrophotometer (SPECTROstar Nano, BMG Labtech GmbH, Ortenberg, Germany) to quantify the antiproliferative effects of treated samples relative to negative controls (cells maintained in medium). Every set of conditions was tested at least in two independent experiments, with five parallel wells. Tested agents were initially screened at 20 and 60 μg/mL for *P. harmala* crude extract obtained from different plant parts, while the isolated bioactive constituents were screened in 10 and 30 µM concentrations. In the case of the tested substances that showed a growth inhibition higher than 60% in most of the tested cancerous cells, the experiment was replicated using a dilution series. Nonlinear sigmoidal dose-response curves were generated from six-point concentration data, and IC_50_ values were determined using GraphPad Prism software (v5.01, GraphPad Software, San Diego, CA, United States). The maximum exposed DMSO concentration (0.6%, vehicle control) exhibited no notable impact on the investigated cells’ viability under the same experimental conditions. Cisplatin, a clinically utilized chemotherapeutic agent, served as the positive control, applied in 0.1–30 µM concentrations.

### 2.8 Hoechst 33258–propidium iodide fluorescent (HOPI) double staining

Hoechst 33258–propidium iodide (HOPI) dual staining was employed to visualize the alterations in cellular morphology induced by harmine (Fritzer-Szekeres et al., 2008). SiHa and UPCI-SCC-154 cells were plated at 200,000 cells per well into 6-well plates and cultured overnight under standard cell culturing conditions. Subsequently, the cells were treated with escalating concentrations of harmine for 24 h. Afterward, the medium in each well was replaced with fresh medium supplemented with Hoechst 33258 (HO) and propidium iodide (PI) in a final concentration of 5 μg/mL and 3 μg/mL, respectively. The stained cells were incubated for 90 min. After replacing the medium in each well, fluorescence images were captured by a Nikon Eclipse TS100 microscope (Nikon Instruments Europe, Amstelveen, Netherlands) supplied with specific filters set for Hoechst and PI visualization. Apoptotic cells were identified by characteristic morphological features such as chromatin condensation and nuclear fragmentation following HO staining, while necrotic cells were identified by PI uptake, indicating loss of membrane integrity.

### 2.9 Flow cytometry

Quantification of DNA content for cell cycle analyses was carried out following previous reports (Alföldi et al., 2019). SiHa and UPCI-SCC-154 cells were plated at 80,000 - 200,000 cells per well. After incubating the plates overnight, cells were exposed to increased concentrations of harmine. After 48 h and 72 h treatment, a sequence of cells' washing, trypsinization, and centrifugation was performed. The collected cells were resuspended in staining solution (10 μg/mL PI, 0.1% Triton-X, 10 μg/mL RNase A, and 0.1% sodium citrate in PBS). DNA content was analyzed using either FACSCalibur (BD Biosciences, San Jose, CA, United States) or CytoFLEX flow cytometer (Beckman Coulter, Brea, CA, United States) for SiHa and UPCI-SCC-154 cells, respectively; in each sample, at least 20,000 events were detected. The distribution of cells across cell cycle phases was analyzed by the Kaluza Analysis software (Beckman Coulter, Brea, CA, United States) or ModFit LT software (v3.3.11, Verity Software House, Topsham, ME, United States). Untreated samples were considered as controls. Furthermore, apoptotic cell populations were determined through Annexin V-Alexa 488 (AnnV)/PI dual staining (Szebeni et al., 2017). Cells were displayed on AnnV (FL1) and PI (FL3), where those exhibiting positivity for AnnV but staining negative for PI were categorized as early apoptotic. Cells positive for both AnnV/PI were indicated as late apoptotic cell populations. After 24 h incubation, 10,000 events were measured by FACSCalibur Flow Cytometer for each sample using CellQuest software (Becton Dickinson, Franklin Lakes, NJ, United States).

### 2.10 Caspase-3 activity measurement

The commercially available fluorimetric caspase-3 kit (CASP3F) was used to explore the proapoptotic activity of harmine, in reference to the earlier report (Smith and Cook, 2004). SiHa and UPCI-SCC-154 cells were distributed in 96-well microplates. After overnight incubation, harmine in escalating concentrations was added to each well. Cisplatin served as a positive apoptosis inducer. Following 72 h of incubation, cells were lysed by adding lysis buffer on ice for approximately 15–20 min. The assay buffer containing the assay kit substrate was added to the cell lysates. Then, 200 µL of each sample cell lysate, assay buffer, and substrate were transferred into a black-sided transparent-bottom 96-well microplate. Fluorescence intensity was measured using a CLARIOstar^Plus^ plate reader (BMG Labtech GmbH, Ortenberg, Germany) set at 360 nm excitation and 460 nm emission wavelength. The assay buffer containing equivalent amounts of reaction substrate was used as a background control. The fold induction in caspase-3 activity was determined by comparing the fluorescence intensity of treated and untreated groups. The assay was repeated twice with three parallels.

### 2.11 Tubulin polymerization assay

Harmine’s effect on tubulin dynamics was determined through a standardized commercial assay reagent set (Cytoskeleton Inc., Denver, CO, United States) based on an adaptation of the original methods (Shelanski et al., 1973; Lee and Timasheff, 1977). Briefly, harmine solutions were dispensed to a pre-warmed 96-well microplate. As recommended by the manufacturer, paclitaxel was included as an assay-positive reference, while a general tubulin buffer served as the assay-negative reference. To initiate the polymerization, tubulin was added to each well. The polymerization reaction was measured for 60 min to monitor the absorbance readings every minute at 340 nm by a microplate reader. The maximum polymerization rate (Vmax) was evaluated based on the mean absorbance values recorded at six sequential time intervals. The peak variation between two consecutive time intervals was taken as the Vmax value. The assay was repeated, with two parallels for each sample.

### 2.12 Wound healing assay

Cell motility was evaluated through the wound healing assay (Behrens et al., 2010). The SiHa, C33A, and UPCI-SCC-154 cells were plated onto 12-well plates fitted with ibidi inserts (ibidi GmbH, Gräfelfing, Germany) at densities of 30,000 cells per insert chamber for SiHa, and 40,000 cells per insert chamber for C33A and UPCI-SCC-154. Escalating concentrations of the investigated substances in a reduced serum culture medium were introduced to the wells. Images were captured at 0, 24, and 48 h after treatment using QCapture Pro software (QImaging, Surrey, British Columbia, Canada). The extent of cell migration was determined by measuring the uncovered regions in the cell monolayer in treated samples compared with untreated control samples using the ImageJ software (National Institutes of Health (NIH), Bethesda, MD, United States). Each experiment was conducted with at least three replicates.

### 2.13 Boyden chamber assay

Cell invasion was evaluated using 24-well transwell inserts equipped with an 8-µm pore PET membrane coated with a thin Matrigel basement matrix layer (Corning^®^ BioCoat™ Matrigel^®^ Invasion Chamber, Bedford, MA, United States), as previously detailed (Albini et al., 1987). Cells were loaded into the upper compartment with a serum-derived medium supplemented with sub-inhibitory concentrations of harmine (less than the IC_50_ value in the 72 h MTT assay). In contrast, the lower compartment was supplemented with either a 10% FBS-enriched medium (SiHa) or 10% FBS-supplied medium and non-cancerous NIH/3T3 fibroblast-conditioned medium mixture (UPCI-SCC-154) as a chemoattractant. After incubating for 24 h or 48 h, cells that successfully invaded were fixed and subsequently stained with 1% crystal violet dye. The invading cells were counted, and a minimum of five microscopic fields per insert were captured using a Nikon Eclipse TS100 microscope. The assay was conducted twice with duplicates for each condition.

### 2.14 Statistical analysis

GraphPad Prism software (version 5.01) was used for all statistical evaluations. Values are presented as average ±SEM based on not less than two distinct experimental replicates. A one-way analysis of variance (ANOVA) coupled with Dunnett’s post-test was used for comparison among groups. Statistical significance was defined relative to control; *p < 0.05, **p < 0.01, ***p < 0.001.

## 3 Results

### 3.1 Alkaloid content of the *P. harmala* extracts

**FIGURE 1:**
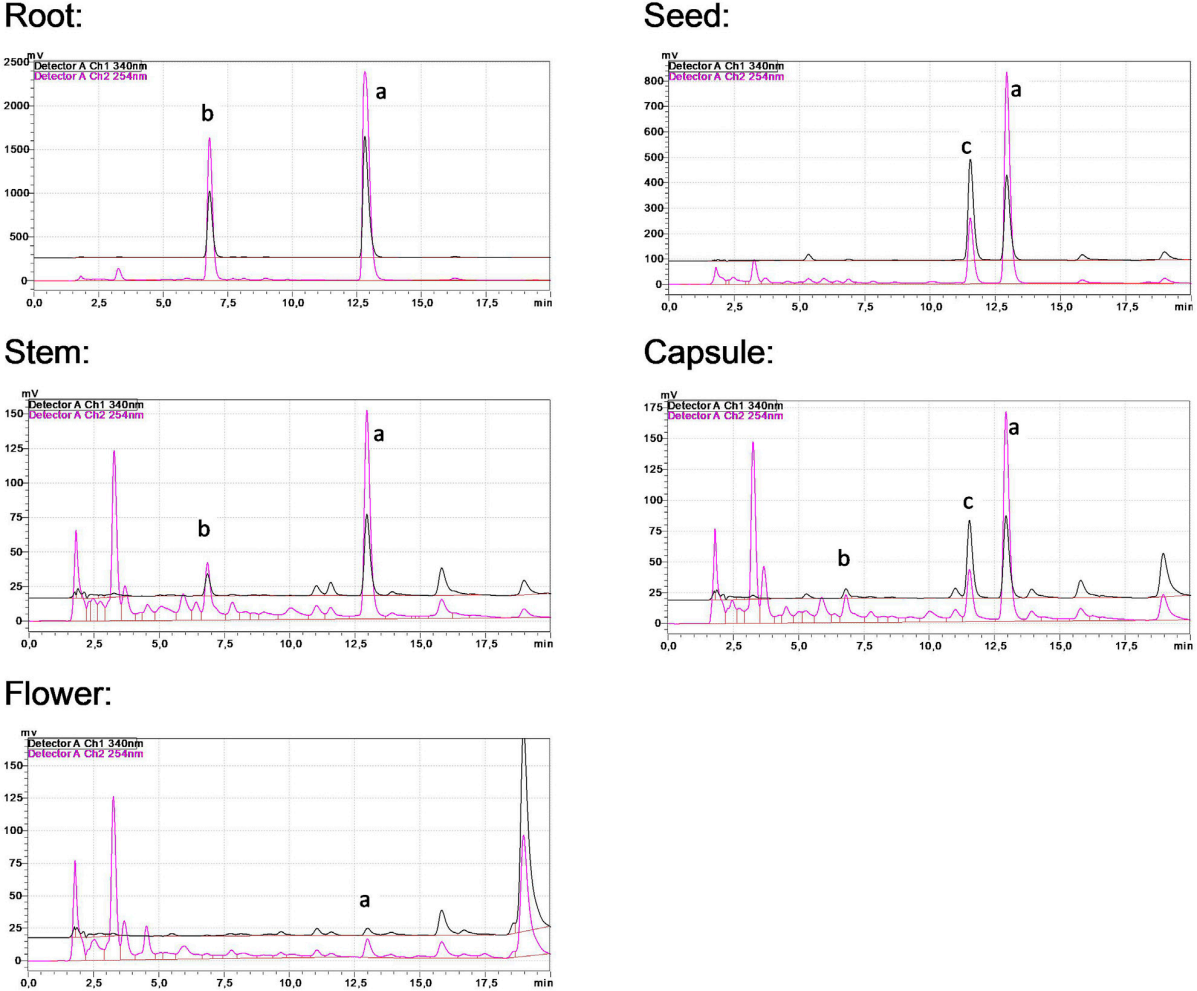
HPLC chromatograms of *P. harmala* extracts. Peaks (a‐c) represent harmine, harmol, and harmaline, respectively.

The levels of the three main alkaloids across different plant sections have been determined using an RP-HPLC procedure (Figure 1; Table 1). Harmine has emerged as the most abundant alkaloid, especially predominant in the root and the seed; it can be regarded as the main component. Substantial harmol content was detected in the root extract, while harmaline was present in all processed organs at considerably lower concentrations.

**TABLE 1:** Alkaloid quantities of *P. harmala* extracts.

Extract	Quantity of the alkaloids in *P. harmala* organs (mg/g ± SD)		
	Harmine	Harmol	Harmaline
Root	151.90 ± 0.27	70.51 ± 0.11	n/d
Seed	35.09 ± 1.41	1.44 ± 0.027	0.495 ± 0.0009
Stem	6.44 ± 0.03	2.32 ± 0.023	0.0125 ± 0.0006
Capsule	7.28 ± 0.01	1.52 ± 0.003	0.0785 ± 0.0001
Flower	0.585 ± 0.037	0.87 ± 0.003	0.0035 ± 0.0002

n/d; not determined.

### 3.2 MTT antiproliferative assay

**FIGURE 2:**
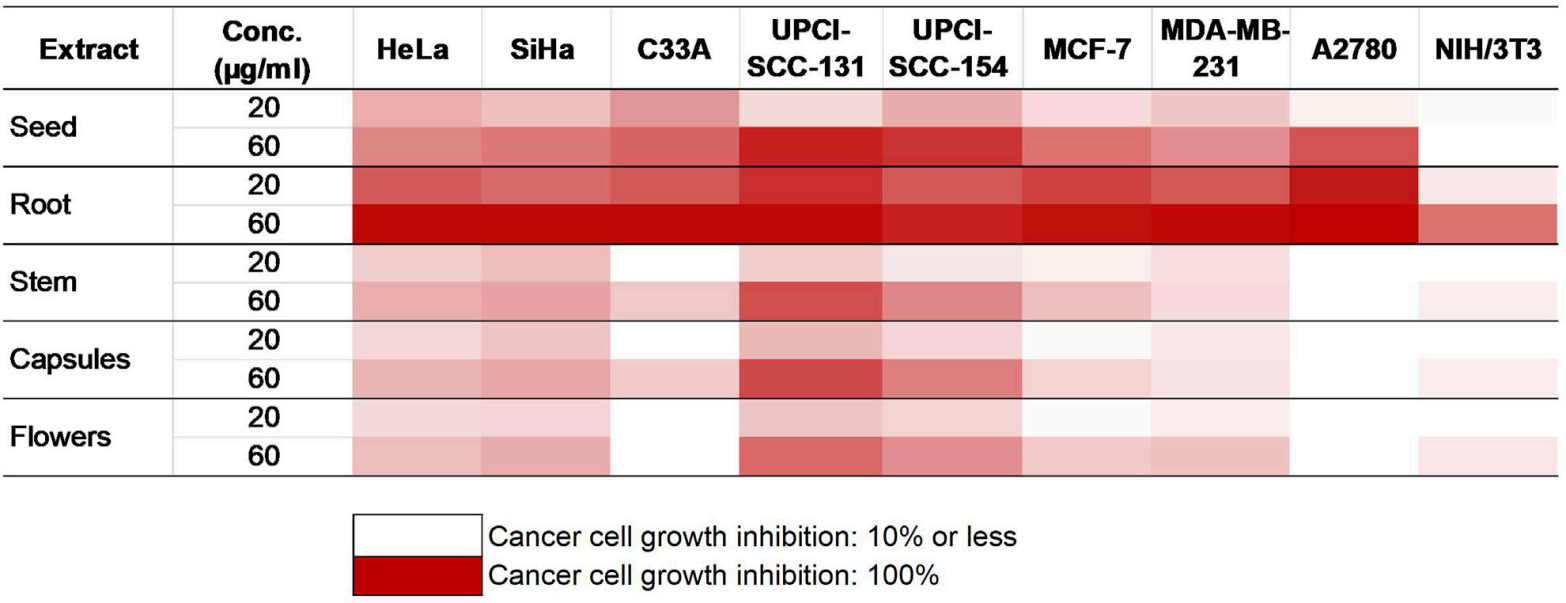
Antiproliferative activities of *P. harmala* extract.

**FIGURE 3:**
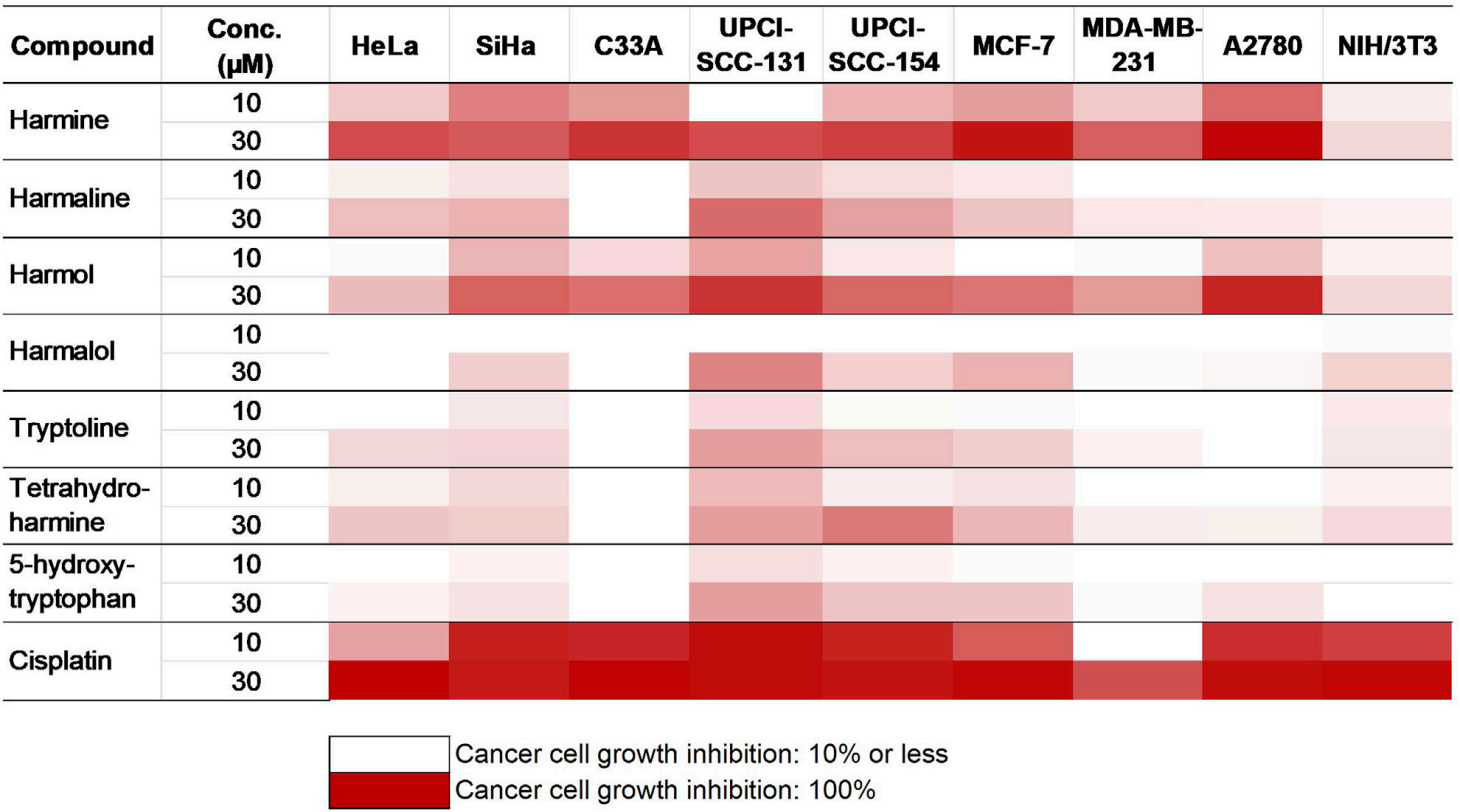
Antiproliferative activities of the isolated alkaloids.

The impact of *P. harmala* on cancer cell growth in selected breast and gynecological cancer cells (MCF-7, MDA-MB-231, HeLa, SiHa, C33A, and A2780) as well as oral carcinoma cells (UPCI-SCC-154 and UPCI-SCC-131) was assessed by treating the cells with escalated doses of the tested substances for 72 h, followed by evaluation by MTT assay. Cell lines were selected based on their HPV infection status or receptor expression. Our data demonstrated that the root extract exhibited the highest inhibitory effect among the extracts from various *P. harmala’s *parts (Figure 2). Similarly, among the isolated β-carboline alkaloids, harmine exerted a pronounced growth-inhibitory effect, as shown in Figure 3 (numerical data for Figures 2, 3 are presented as Supplementary Tables S1, S2, respectively, in the Supplementary Material). In instances where tested substances significantly impaired cell proliferation, IC_50_ values were determined by fitting nonlinear sigmoidal dose-response curves (Table 2; Supplementary Figures S12–S14). Generally, harmine was the most effective compound among the isolated alkaloids, with the lowest IC_50_ value of 6.05 µM. Moreover, the results showed no apparent cytotoxic effects in NIH/3T3 murine fibroblast cells, indicating its considerable tumor selectivity. Based on the findings, harmine was chosen for subsequent *in vitro* investigations on cervical cancer (SiHa) and oral SCC (UPCI-SCC-154) cell lines.

**TABLE 2:** Calculated IC_50_ values for the most active compounds.

Cell line	Calculated IC_50_ value ±SEM			
	µg/mL	µM		
	Root	Harmine	Harmol	Cisplatin
HeLa	26.74 ± 3.66	16.56 ± 0.74	>30	12.43 ± 0.15
SiHa	8.47 ± 0.48	6.05 ± 0.52	11.89 ± 2.65	4.80 ± 0.51
C33A	12.95 ± 1.87	12.27 ± 1.65	21.50 ± 0.83	4.70 ± 1.16
MCF-7	9.86 ± 0.67	8.06 ± 1.70	20.50 ± 1.80	8.19 ± 0.14
MDA-MB-231	13.65 ± 0.93	18.77 ± 3.34	>30	19.12 ± 0.02
A2780	4.87 ± 1.09	6.54 ± 0.17	13.40 ± 0.83	1.34 ± 0.04
UPCI-SCC-154	10.83 ± 0.88	13.30 ± 0.57	22.59 ± 0.24	1.29 ± 0.001
UPCI-SCC-131	23.13 ± 2.30	27.85 ± 0.50	>30	1.37 ± 0.15
NIH/3T3	56.43 ± 0.32	>30	>30	5.11 ± 0.38

### 3.3 Apoptosis induction

**FIGURE 4:**
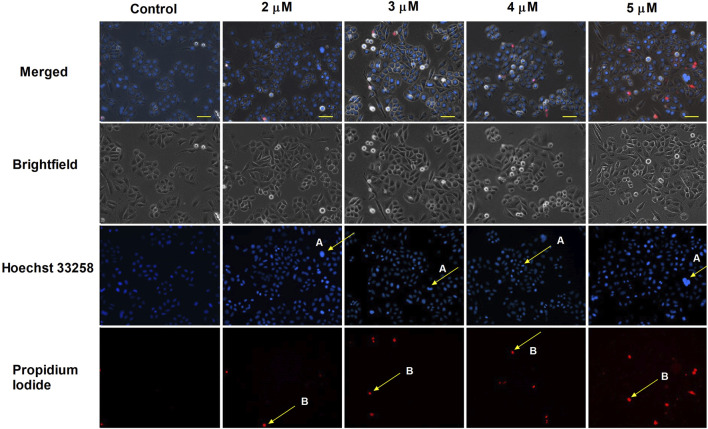
Harmine-induced apoptosis in SiHa cells was assessed by Hoechst 33258 (blue fluorescence) and propidium iodide (red fluorescence) after 24 h of treatment. Apoptotic (arrow A) and necrotic (arrow B) cell nuclei after exposure to different concentrations of harmine was visualized by a fluorescence microscope. The bar in the pictures indicates 100 µm.

**FIGURE 5:**
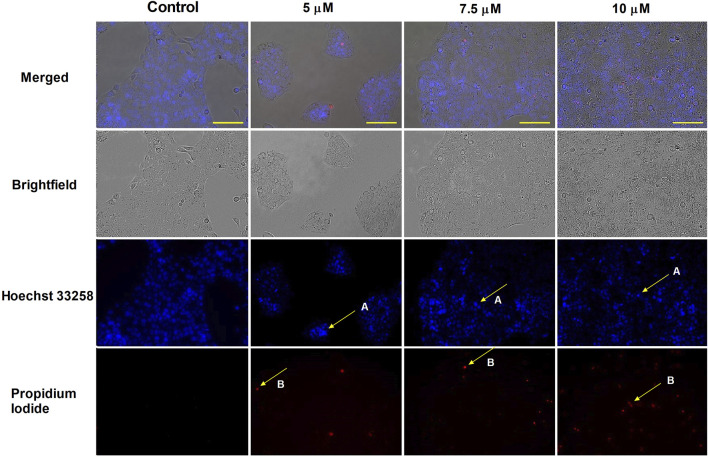
Harmine-induced apoptosis in UPCI-SCC-154 cells was assessed by Hoechst 33258 (blue fluorescence) and propidium iodide (red fluorescence) after 24 h of treatment. Apoptotic (arrow A) and necrotic (arrow B) cell nuclei after exposure to different concentrations of harmine was visualized by a fluorescence microscope. The bar in the pictures indicates 100 µm.

**FIGURE 6:**
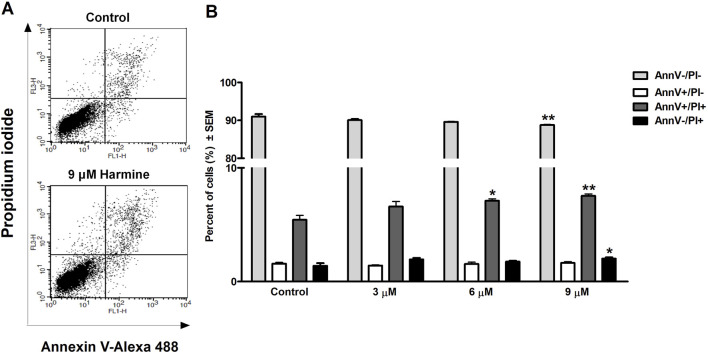
Harmine-induced late apoptosis in SiHa cells after 24 h of incubation. Representative dot plots **(A)** and the percentage of apoptotic cells **(B)** was quantified and distinguished using cell staining with Annexin V-Alexa 488–PI. The numbers of viable (AnnV-/PI-), early apoptotic (AnnV+/PI-), late apoptotic (AnnV+/PI+), and necrotic (AnnV-/PI+) populations are given as percentages. Data are presented as mean ± SEM. * and ** indicate P < 0.05 and P < 0.01, respectively, compared to control.

HOPI staining was used to identify apoptotic morphological changes. After 24 h of treating SiHa (2, 3, 4, and 5 µM) and UPCI-SCC-154 (5, 7.5, and 10 µM) with harmine, images from the same field were acquired under different optical filters. Our results showed that cells displayed typical features of early apoptosis, as shown in Figures 4, 5, respectively. Further, late apoptosis or necrosis was evident as the cells’ nuclei were stained red, indicating propidium iodide penetration, thus suggesting possible damage to the cell membranes. For further proof of the proapoptotic effect of harmine, the Annexin V-Alexa 488–propidium iodide dual staining kit was used and analyzed via flow cytometry. A concentration-dependent increase in the early and late apoptotic SiHa cell populations from 7.1% to 13.8% was observed following 24 h of harmine exposure (Figure 6).

### 3.4 Cell cycle analysis

**FIGURE 7:**
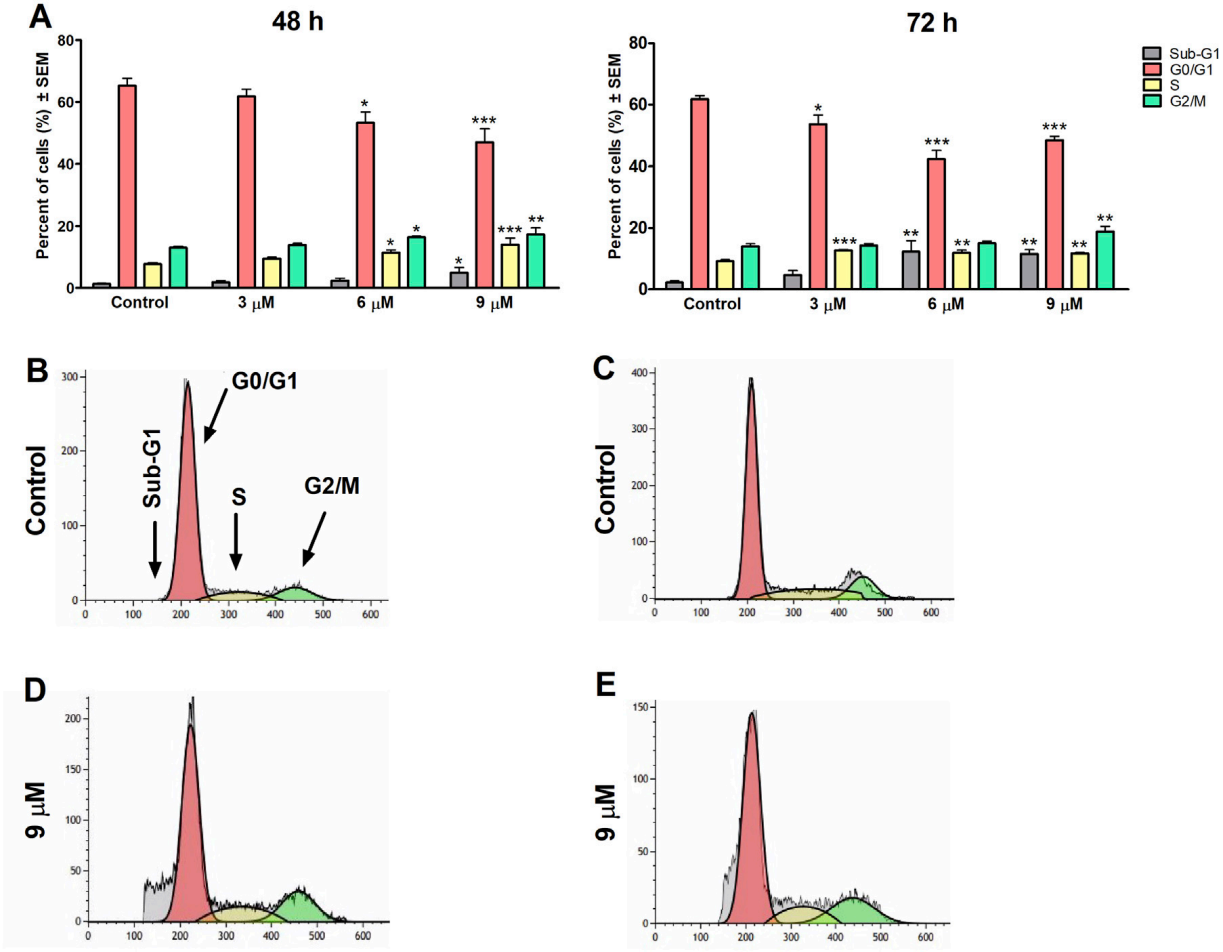
Harmine increases the sub-G1 population and induces cell cycle arrest in the S and G2/M phases in the SiHa cells, as detected 48 h and 72 h post-treatment **(A)**. Data are presented as mean ± SEM. *, ** and *** indicate P < 0.05, P < 0.01 and P < 0.001, respectively, compared to control. Data are from two independent experiments performed in triplicate. Representative histograms: Controls **(B,C)** 48 h and 72 h post-treatment, respectively, and treated cells **(D,E)** 48 h and 72 h post-treatment, respectively.

**FIGURE 8:**
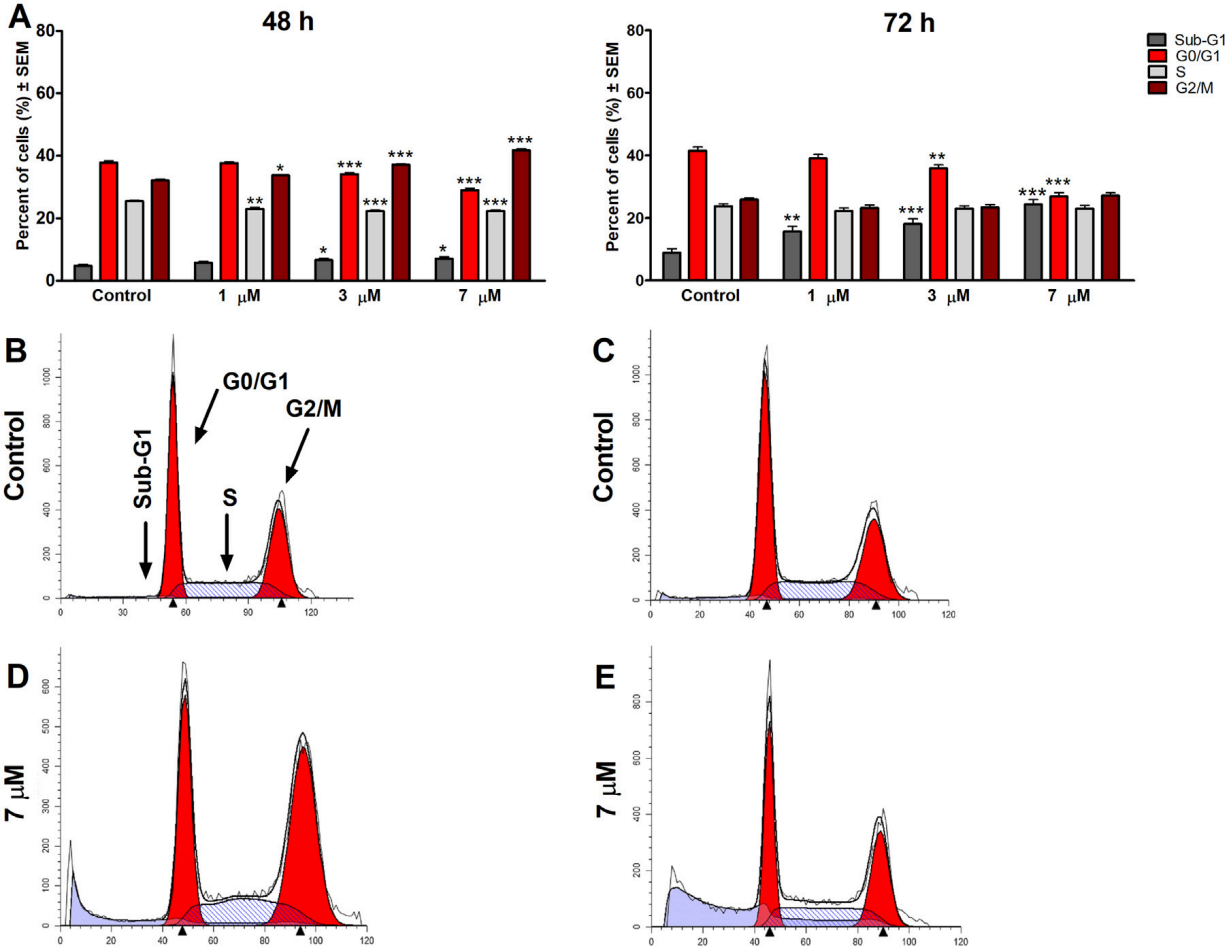
Harmine induces cell cycle arrest in the G2/M phase 48 h post-treatment and increases the sub-G1 population in the UPCI-SCC-154 cells, as detected 48 h and 72 h post-treatment **(A)**. Data are presented as mean ± SEM. *, ** and *** indicate P < 0.05, P < 0.01 and P < 0.001, respectively, compared to control. Data are from two independent experiments performed in triplicate. Representative histograms: Controls **(B,C)** 48 h and 72 h post-treatment, respectively, and treated cells **(D,E)** 48 h and 72 h post-treatment, respectively.

To clarify harmine’s possible mechanism of antiproliferative effects, its impact on the cell cycle distribution of SiHa and UPCI-SCC-154 cells was assessed. Flow cytometry was utilized to analyze cell populations after the cells were treated with or without harmine. The treatment concentrations were selected according to the previously determined IC_50_ values. Our results revealed that after 48 h and 72 h of harmine’s exposure, SiHa cells demonstrated S and G2/M phases arrest, with concurrent decline in the G1 phase (Figure 7). Besides that, the sub-G1 peak was increased significantly after harmine treatment, more clearly seen after longer incubation of 72 h. These changes occurred in a dose-related manner. With consistent experimental settings, the oral cancer cells, UPCI-SCC-154 cells, treated with harmine showed concentration-dependent G2/M arrest and S phase depletion with a decreased percentage of the G1 phase, observed 48 h following harmine exposure. Extending incubation to 72 h led to a concentration-dependent increase in the sub-G1 population, alongside a marked reduction in the G1 population (Figure 8). Representative dot plots for Figures 7, 8 are presented as Supplementary Figures S15, S16, respectively, in the Supplementary Material.

### 3.5 Evaluation of caspase-3 activity

**FIGURE 9:**
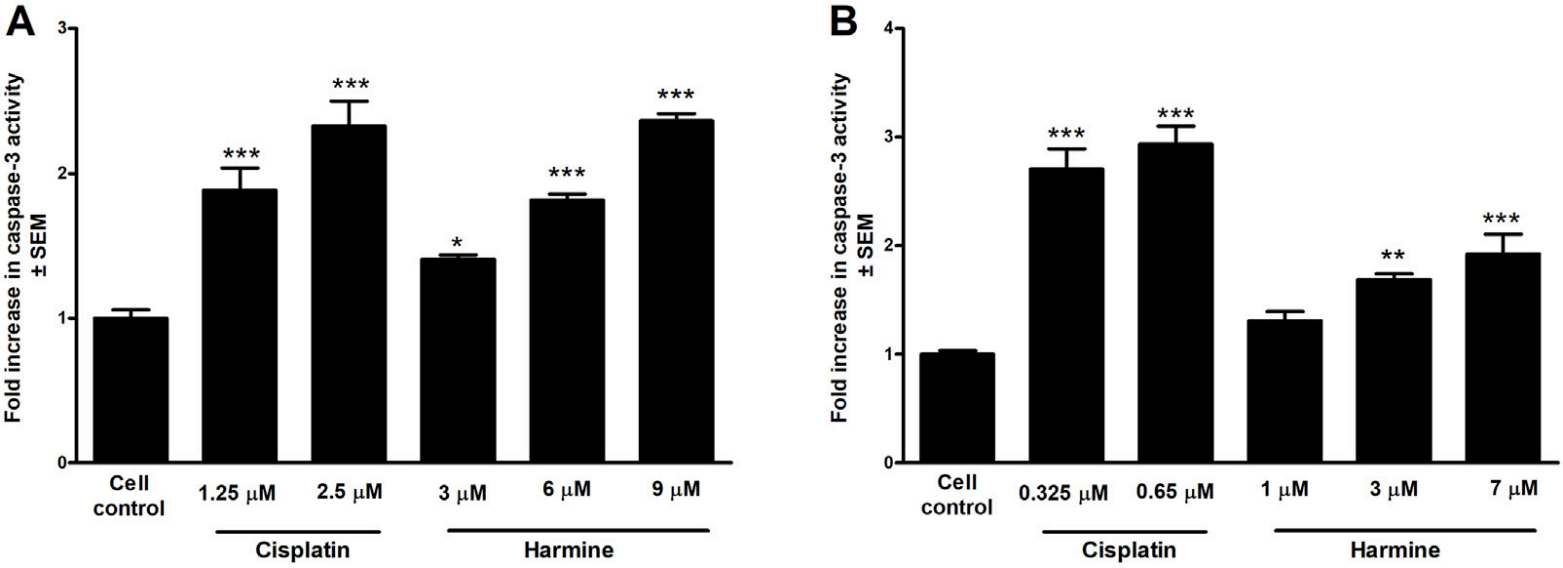
Harmine enhances the caspase-3 activity of the tested HPV-positive cancer cells. The results show the fold increases of the caspase-3 activity in **(A)** cervical cells SiHa and **(B)** oral squamous carcinoma UPCI-SCC-154 cells compared to the untreated control, 72 h post-harmine exposure. Data are presented as mean ± SEM. *, ** and *** indicate P < 0.05, P < 0.01 and P < 0.001, respectively, compared to control samples.

Caspase-3 activity, a key executor of apoptosis, was measured after an incubation of 72 h with harmine using a fluorometric method. Our data revealed a dose-dependent elevation in caspase-3 enzymatic function in both tested cell lines, showing a trend comparable to that of cisplatin, a known caspase-3 inducer. Noteworthy, the enzyme activity was more pronounced in harmine-treated UPCI-SCC-154 cells than in the cervical SiHa cells, which aligns with the flow cytometry findings (Figure 9).

### 3.6 Tubulin polymerization

**FIGURE 10:**
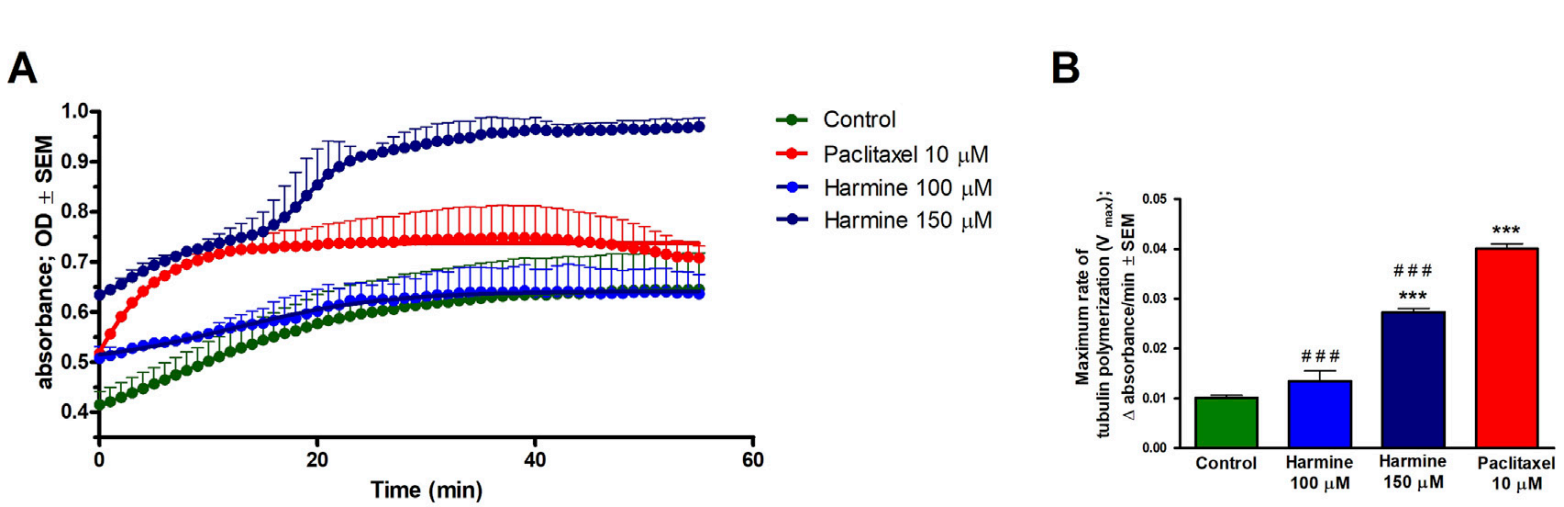
Harmine accelerates tubulin polymerization using a cell-free tubulin polymerization assay. Tubulin was incubated with the general tubulin buffer and paclitaxel as negative and positive controls, respectively. **(A)** Tubulin polymerization curve plotted using GraphPad Prism 5.01 software. **(B)** Maximum rate of tubulin polymerization. Each condition was performed in duplicate at two separate measurements. Data presented are mean ± SEM, *** indicates P < 0.001, compared to negative control samples, and ### indicates P < 0.001, compared to paclitaxel.

Based on the noted G2/M phase cell cycle arrest, harmine’s effect on the dynamics of microtubules was evaluated through a standard tubulin polymerization model. Following the supplier’s protocol and considering the calculated inhibitory concentrations, we applied 100 and 150 µM of harmine, while paclitaxel served as the positive control. The kinetic curves indicate that the higher applied concentration of harmine significantly augmented tubulin assembly, which was described by an elevated maximum polymerization rate (Vmax) compared to the untreated control (Figure 10). However, these changes were lower than those observed with the reference positive control, paclitaxel.

### 3.7 Wound-healing assay

**FIGURE 11:**
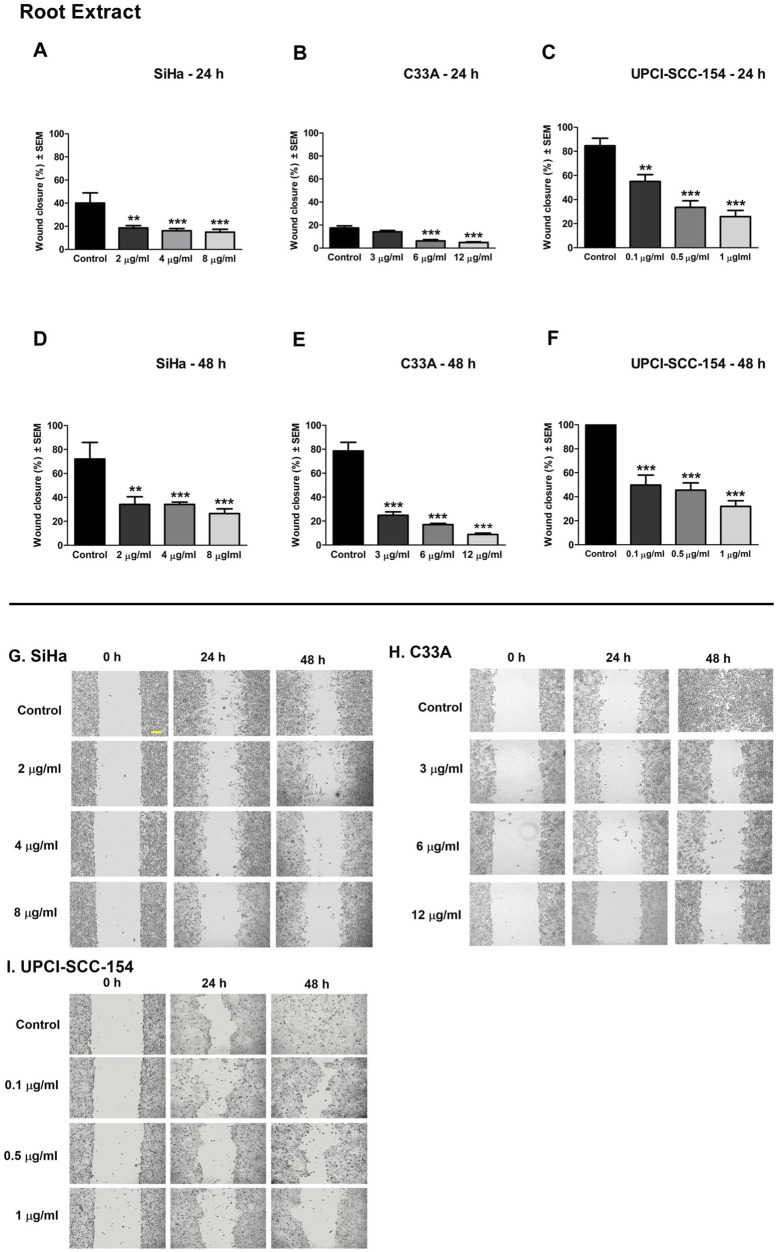
*P. harmala* root extract significantly suppresses the migration of different HPV-status cancer cells. Upper panels: Graphs show the percentage of cell-free area at 24 h and 48 h post-treatment in **(A–D)** SiHa, **(B–E)** C33A, and **(C–F)** UPCI-SCC-154 cells compared to the control. Lower panels **(G,H,I)**: representative images of the suppressed cell migration at 0 h, 24 h, and 48 h for SiHa, C33A, and UPCI-SCC-154 cells. The bar in the pictures indicates 100 μm. Data presented are mean ± SEM of at least twice independent measurements in triplicate. ** and *** indicate P < 0.01 and P < 0.001, respectively, compared to control samples.

**FIGURE 12:**
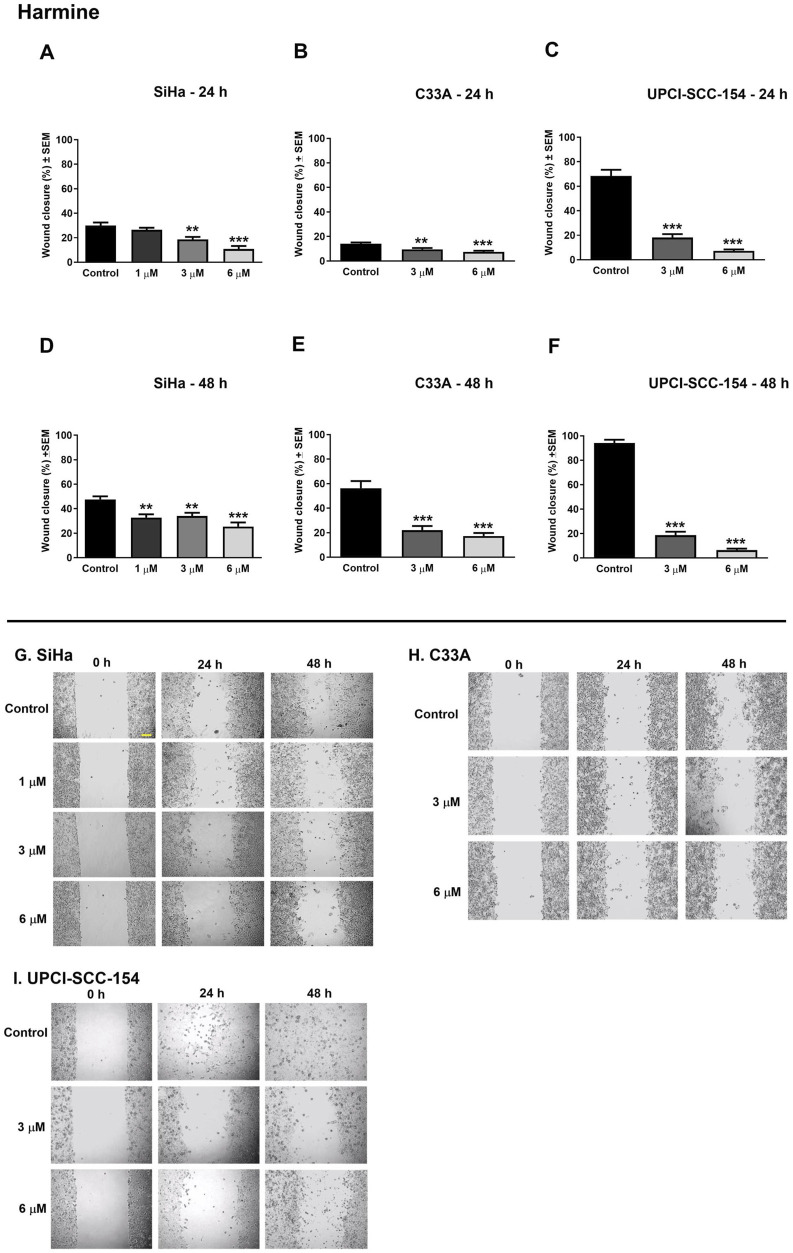
Harmine significantly suppresses the migration of different HPV-status cancer cells. Upper panels: Graphs show the percentage of cell-free area at 24 h and 48 h post-treatment in **(A–D)** SiHa, **(B–E)** C33A, and **(C–F)** UPCI-SCC-154 cells compared to the control. Lower panels **(G,H,I)**: representative images of the suppressed cell migration at 0 h, 24 h, and 48 h for SiHa, C33A, and UPCI-SCC-154 cells. The bar in the pictures indicates 100 μm. Data presented are mean ± SEM of at least twice independent measurements in triplicate. ** and *** indicate P < 0.01 and P < 0.001, respectively, compared to control samples.

Cell motility plays an essential role during cancer metastasis. The root extract has been employed as a complementary traditional therapy in various regions across the world (Moloudizargari et al., 2013; Shahrajabian et al., 2021), and our results proved its potential antiproliferative effect against the tested human cancer cell lines. Therefore, we performed a wound healing assay using cell lines of different origins and HPV infection statuses: the cervical carcinoma cells (HPV 16-positive SiHa and HPV-negative C33A) and the HPV 16-positive oral squamous cell carcinoma cells UPCI-SCC-154 to assess the effect of root extract on cancer cell motility. In addition, harmine’s antimigratory effect has been investigated using the same method. The cell migration was quantified at 0, 24, and 48 h after treatment by analyzing wound closure from image fields. As shown in Figures 11, 12, the examined substances markedly inhibited the cell migration of the cells mentioned above, even at sub-inhibitory concentrations.

### 3.8 Boyden-chamber assay

**FIGURE 13:**
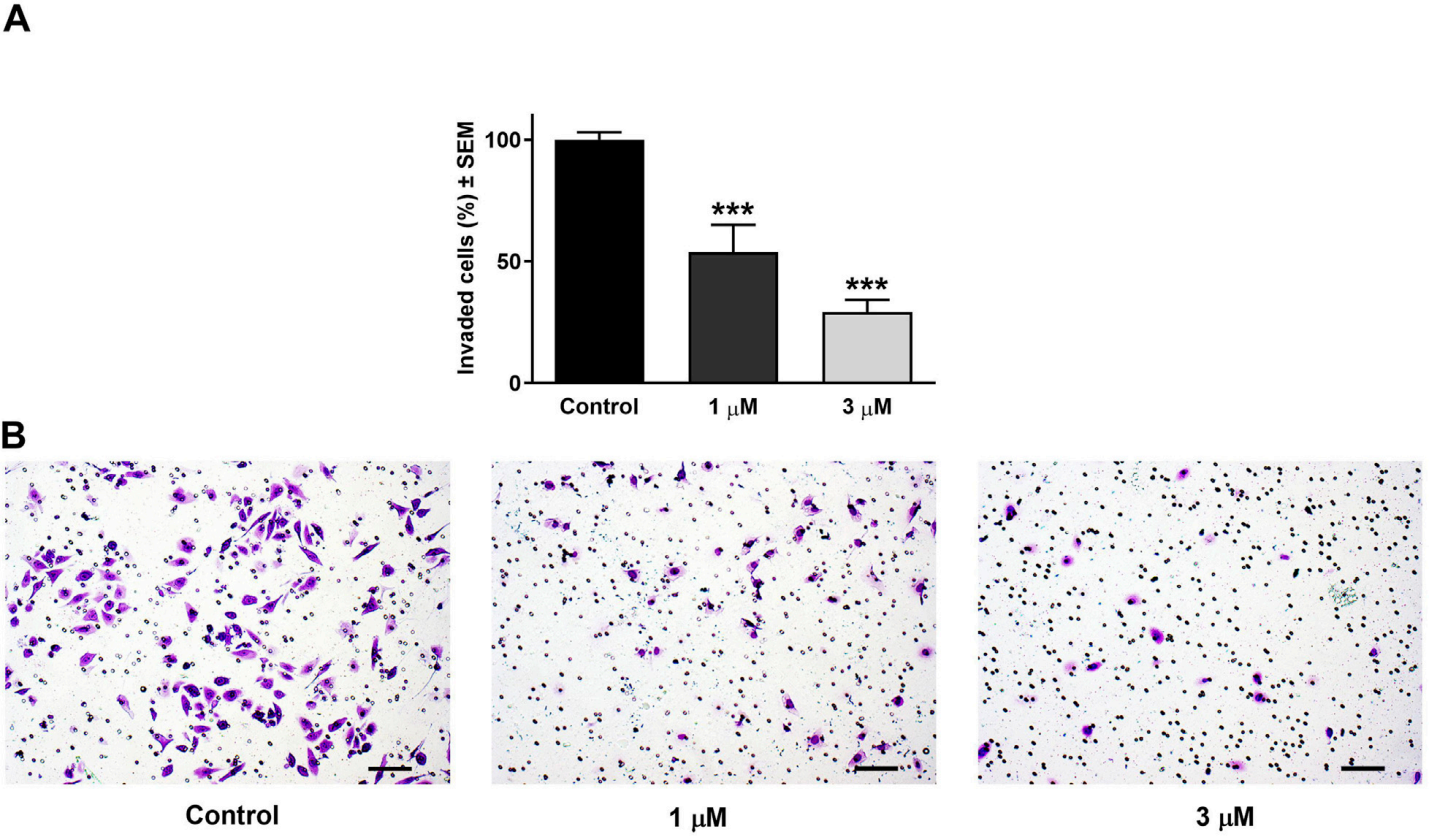
Harmine reduced cell invasion in Matrigel-coated Boyden chambers. **(A)** Percentages of invaded SiHa cells 24 h post-treatment with different concentrations of harmine. **(B)** Representative images illustrate the test substance’s anti-invasive ability on SiHa cells. The bar in the pictures indicates 100 µm. Data are presented as mean ± SEM of two independent measurements with a duplicate. *** indicates P < 0.001, compared to control samples.

**FIGURE 14:**
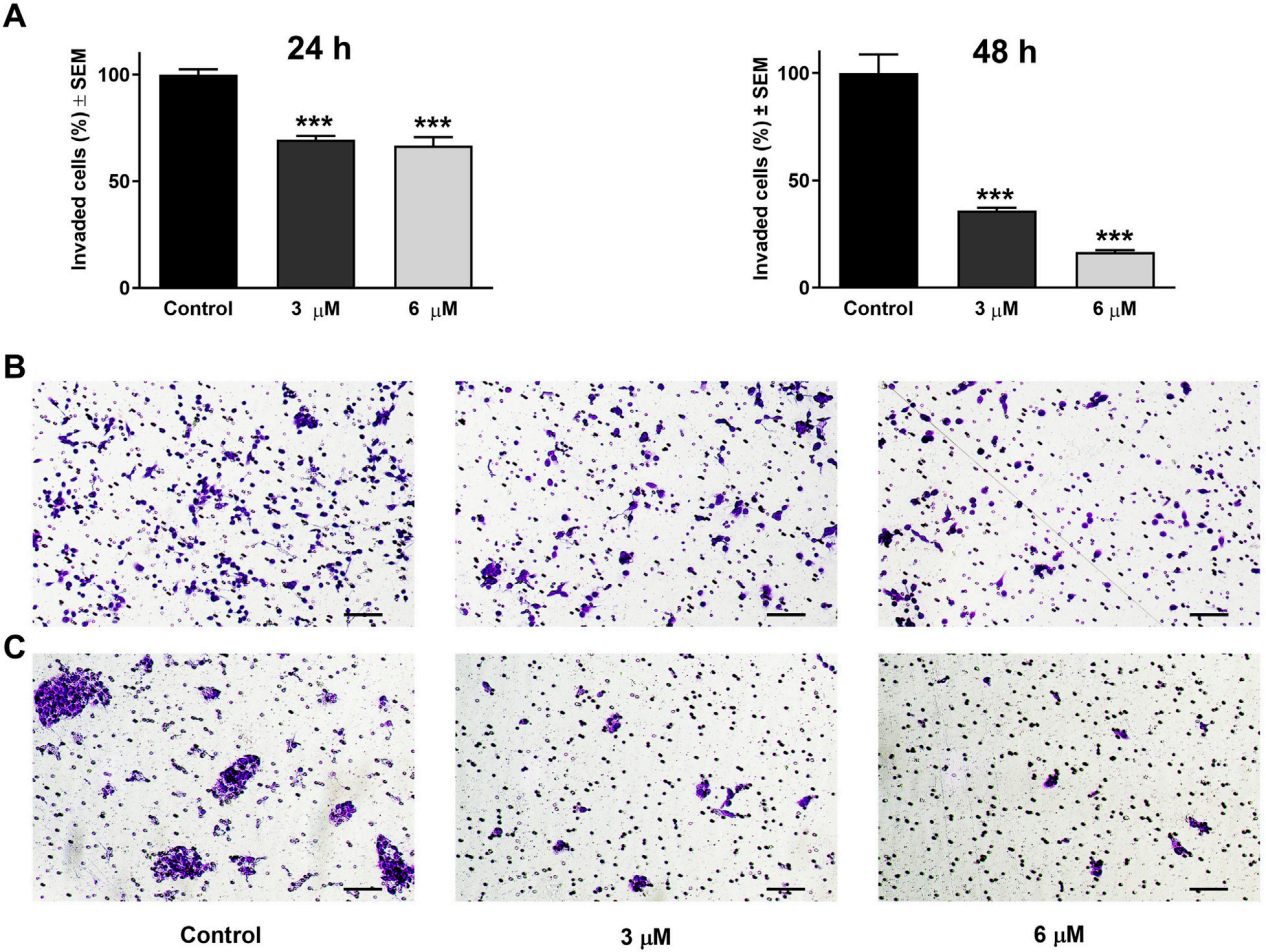
Harmine reduced cell invasion in Matrigel-coated Boyden chambers. **(A)** Percentages of invaded UPCI-SCC-154 cells 24 h and 48 h post-treatment with different concentrations of harmine. **(B,C)** Representative images illustrate the test substance’s anti-invasive ability on UPCI-SCC-154 cells 24 h and 48 h post-treatment, respectively. The bar in the pictures indicates 100 μm. Data are presented as mean ± SEM of two independent measurements with a duplicate. *** indicates P < 0.001, compared to control samples.

Beyond its effect on cell motility, the impact of harmine on the invasion potential of cancer cells was evaluated through a Boyden chamber coated with a Matrigel layer. The SiHa and UPCI-SCC-154 cells were incubated with varying concentrations of harmine for 24 and 24 or 48 h, respectively. The findings indicated that harmine reduced the invasion capacity of both cell types compared with the untreated controls. A significant reduction in invaded cells was observed 24 h post-treatment, with increasing concentration of harmine in cervical SiHa cells (Figure 13). However, in UPCI-SCC-154 cells, the concentration-dependent effect was revealed after a more prolonged incubation period (Figure 14). These results confirm that harmine has anti-invasive properties, even at concentrations lower than those required for its antiproliferative effect.

## 4 Discussion

The contribution of bioactive natural substances to antineoplastic development is consistently expanding. Due to their diverse chemical structure, bioactive compounds offer promising leads for the discovery of innovative therapeutic agents that could overcome cancer management challenges such as drug resistance and chemotherapy-related side effects. Moreover, more than 60% of contemporary anticancer medicines have originated or been inspired by natural sources (Asma et al., 2022). Several naturally derived alkaloids and diterpenes, including vincristine, vinblastine, camptothecin, and taxanes have been successfully used as anticancer agents (Demain and Vaishnav, 2011). Previously, *P. harmala* has been adopted as a folk remedy for its potential anticancer properties (Lamchouri et al., 2000). The antitumor effects of harmine have been previously reported from experimental research (Zhao and Wink, 2013; Sun et al., 2015; Gao et al., 2017; Ruan et al., 2017; Ding et al., 2019; Nafie et al., 2021). However, according to our knowledge, there is no report about the mechanistic insights into harmine’s activity in cervical and HN cancers.

This study highlighted the possible antitumor properties of *P. harmala* crude extracts and isolated alkaloid constituents on a range of adherent cancer cells. Our data revealed that the root extract exhibited the highest growth inhibitory effect among *P. harmala* total extracts collected from distinct anatomical sections. Harmine, occurring in the root extract in the highest concentration (151.90 ± 0.27; mg/g ± SD) was the most active among the isolated β-carboline alkaloids. Furthermore, harmine displayed an antiproliferative activity on tested cancer cells of breast, gynecological, and HN origins, and minimal activity towards NIH/3T3 cells, indicating high tumor selectivity. The HPV-16-infected cervical (SiHa) and oral (UPCI-SCC-154) cancer cell lines were chosen for additional investigations, aiming to elucidate harmine’s underlying mechanism. Multiple applied *in vitro* methods, including HOPI double staining, AnnV-Alexa488 and PI staining, caspase-3 induction, and evaluation of cell cycle phases, revealed that harmine-treated cancer cells induced apoptosis. Following HOPI double staining of HPV-positive cervical cancer cells and oral squamous carcinoma cells, our data indicated that harmine efficiently triggered both early and late apoptotic events following 24 h of incubation. Apoptosis is typically marked by certain morphological features, including nuclear condensation, formation of membrane blebs, DNA cleavage, and cellular uptake of vital dyes, such as propidium iodide (Martin et al., 1995). Harmine-treated cells exhibited some hallmark features of apoptosis in a dose-dependent manner. Moreover, AnnV-Alexa488/PI dual labeling was conducted. A significant elevation of late apoptotic cell populations was observed in SiHa cervical cancer cells after 24 h incubation with harmine, without a notable elevation in the early apoptosis. Furthermore, the hypodiploid sub-G1 populations increased time- and concentration-dependently after treatment with harmine in both tested cervical and oral squamous carcinoma cells. Quantifying sub-G1 DNA content serves as a widely accepted apoptosis indicator. Caspase-3 is a pivotal cysteine protease enzyme in the cellular apoptosis cascade. Our findings demonstrated significant treatment-enhanced activation of caspase-3 in SiHa and UPCI-SCC-154 cell lines. These findings imply that harmine’s antiproliferative effects might be a consequence of its apoptosis-promoting effects.

To better understand how harmine exerted its antiproliferative effects, cell cycle distributions were analyzed on SiHa cells after 48 h and 72 h of harmine exposure. A marked accumulation of the S and G2/M phases cell population was observed, along with a reduction of the G1 population following a 48 h exposure to 9 µM harmine. At the same time, minimal blockage was detected at a lower 6 µM concentration. However, these changes were further intensified after 72 h of exposure. On the other hand, the HPV-positive oral cell line UPCI-SCC-154 exhibited a greater sensitivity to harmine exposure, even at a low concentration of 1 μM, where harmine exerted noticeable cell cycle disruption after 48 h, including G2/M phase elevation and S phase depletion. With longer incubation, these changes appeared as apoptotic cells, accompanied by a concurrent decline in the G1 phase in a concentration-dependent manner. These findings align with earlier studies reporting harmine-induced G2/M cell cycle phase arrest. For instance, Dai et al. showed that HUVECs exposed to harmine exhibited cell blockage at G2/M (Dai et al., 2012). Likewise, studies demonstrated that harmine exerted cell cycle inhibition at the S and G2/M populations in hepatocellular (Cao et al., 2011) and colon (Liu et al., 2016) cancer cell lines. On the contrary, other studies found G1 phase cell cycle elevation in leukemic and melanoma cancer cells following harmine treatment (Hamsa and Kuttan, 2011a; Oodi et al., 2017). Previous studies have reported that harmine induces apoptosis via the mitochondrial pathway by modulating the Bcl-2/Bax ratio and efficiently downregulating Bcl-2, p-AKT, p-mTOR, and p-E, while upregulating Bax, caspase-3, caspase-8, caspase-9, and Bid. In addition, harmine’s antiangiogenetic property has been documented (Joshi et al., 2025). These observations indicate that harmine can activate both intrinsic and extrinsic apoptotic pathways. Although our study did not directly assess these molecular targets, our results are in line with the literature suggesting that harmine exerts anticancer effects through multiple signaling mechanisms.

Notably, in our study, the harmine concentration used of 1–30 µM for the antiproliferative assay, is relatively high compared to reported plasma levels of harmine. Available pharmacokinetic data are scarce, but a human study shows maintainable plasma concentrations lower than 10 µM post-injection with a rapid decline (Slotkin et al., 1970; Berlowitz et al., 2022). These data suggest that the tested concentrations used in mechanistic experiments (1–9 μM) are closer to physiologically achievable levels. At the same time, high doses in antiproliferative investigations may not be directly translatable to clinical use and carry potential safety concerns (Ables et al., 2024).

Microtubules are essential in key cellular functions, including mitosis and cell division, intracellular transport, and cell migration (Etienne-Manneville, 2013; Akhmanova and Steinmetz, 2015). To assess harmine’s impact on tubulin assembly, a cell-free photometric assay was conducted to monitor tubulin polymerization. Paclitaxel was included as a positive reference control, given its well-established role in promoting tubulin polymerization. Our results indicated that harmine notably accelerated the microtubule formation rate and augmented tubulin assembly in a manner dependent on its utilized concentration. However, the effects observed with harmine were less pronounced than those induced by the widely recognized microtubule stabilizer, paclitaxel. Harmine inhibition of microtubule polymerization suggests potential as an anticancer agent by disrupting mitosis; such activity may also be associated with dose-limiting neurotoxicity, which should be considered in therapeutic development.

Metastatic spread and invasion represent key features of cancer progression (Hanahan and Weinberg, 2000). In many cancer patients, metastases may appear before the primary tumor becomes clinically evident, implying early dissemination of the cancer cells (Cao, 2005). As the motility of cancer cells is a fundamental step in the formation of metastatic lesions, it presents an efficient target for pharmacological intervention to suppress tumor cell dissemination. Since no study explored harmine’s antimetastatic effects in cervical and HN cancers, we investigated harmine-mediated suppression of cell motility and invasion using simplified experimental approaches (wound healing and Boyden chamber *in vitro* assays). Our findings revealed that harmine treatment led to a concentration-dependent reduction in wound closure for SiHa, C33A, and UPCI-SCC-154 cells. Similarly, the root extract significantly suppressed the migratory capacity of these cells in a dose-responsive manner. Furthermore, the invaded cells decline concurrently with increasing harmine concentrations in SiHa and UPCI-SCC-154 cell lines. Findings from both experiments revealed that harmine effectively impedes cell motility in a manner proportional to the applied dose, suggesting the antimetastatic effect of harmine. Since microtubule dynamics are fundamental for cancer cell migration and invasion, disruption of tubulin function can limit metastatic progression. Therefore, the observed effects of the compound may be explained, at least in part, by its ability to interfere with tubulin polymerization (Čermák et al., 2020). Harmine’s antimetastatic effects have also been investigated in distinct cancer cells (Gao et al., 2017; Ruan et al., 2017).

## 5 Conclusion

This study demonstrates that *P. harmala* extracts and alkaloids strongly inhibited cell proliferation and metastasis. Due to its remarkable anticancer activity and tumor selectivity, harmine was explored to determine its possible mechanistic basis in HPV-positive cervical and HN cells. It effectively inhibited the proliferation of the cancer cells, induced cellular death, and disrupted the cell cycle distribution, likely by promoting the stabilization of tubulin polymers. Moreover, it exerted profound antimigratory and anti-invasive properties. These findings provide additional insight into harmine’s antineoplastic activities on cervical and HN cancer cell lines, indicating its potential for further preclinical investigation. However, this study is limited to *in vitro* experiments, and pathway or protein-targeting analyses were not investigated. Additional studies, including animal models and mechanistic investigations, will be needed to further explore its development as a natural therapeutic agent.

## Data Availability

The original contributions presented in the study are included in the article/Supplementary Material, further inquiries can be directed to the corresponding authors.
