# Supplementary material for: Potential anticancer effects of Peganum harmala in human papillomavirus-related cervical and head and neck cancer cells
Source: Front Pharmacol. 2025 Nov 17;16:1668827. doi: 10.3389/fphar.2025.1668827 (PMC12665789; doi:10.3389/fphar.2025.1668827)
Supplement: Supplementary file 1 [file DataSheet1.docx]

Supplementary Material


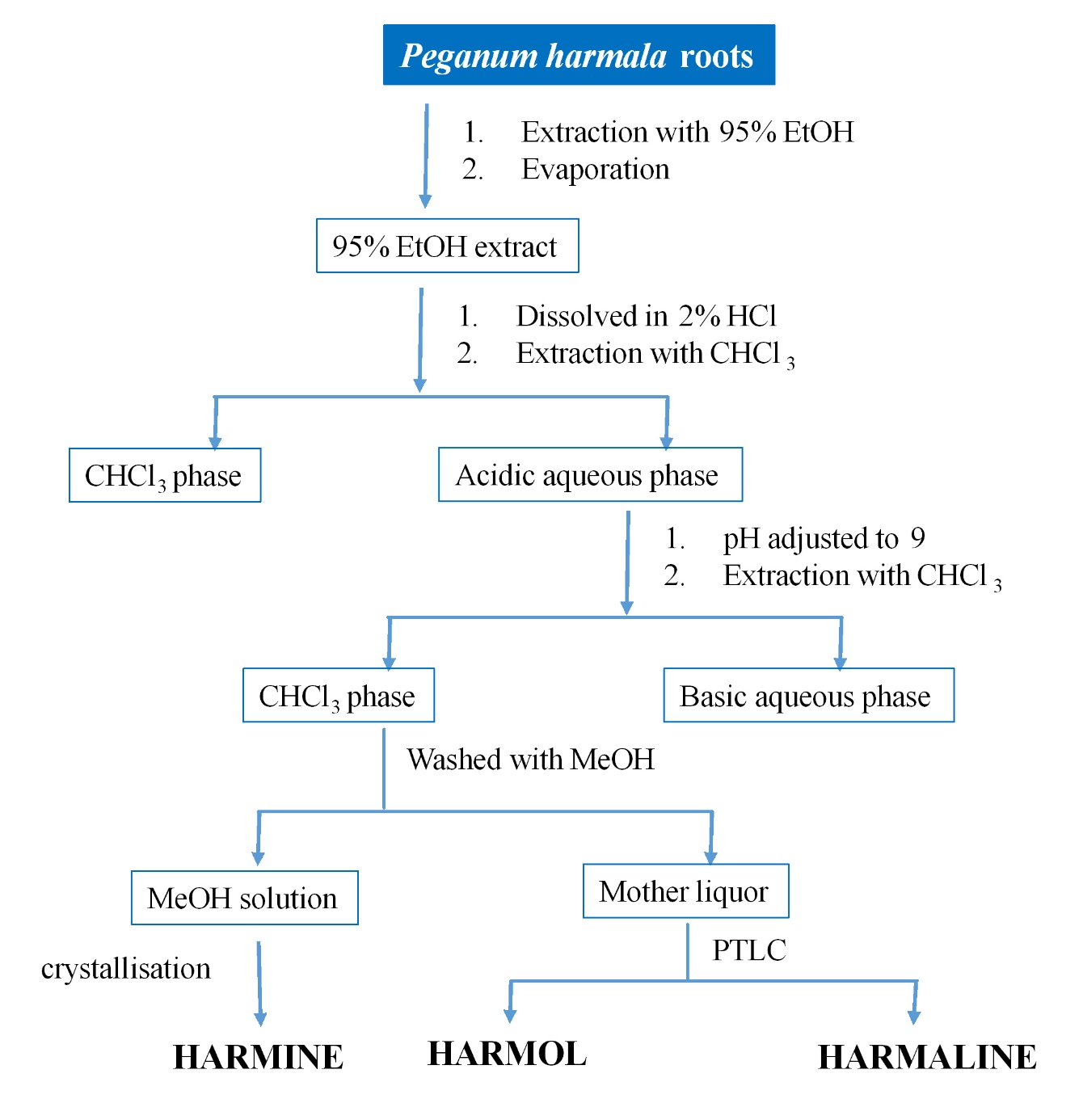


FIGURE S1. Scheme of isolation of alkaloids from *P. harmala* roots.


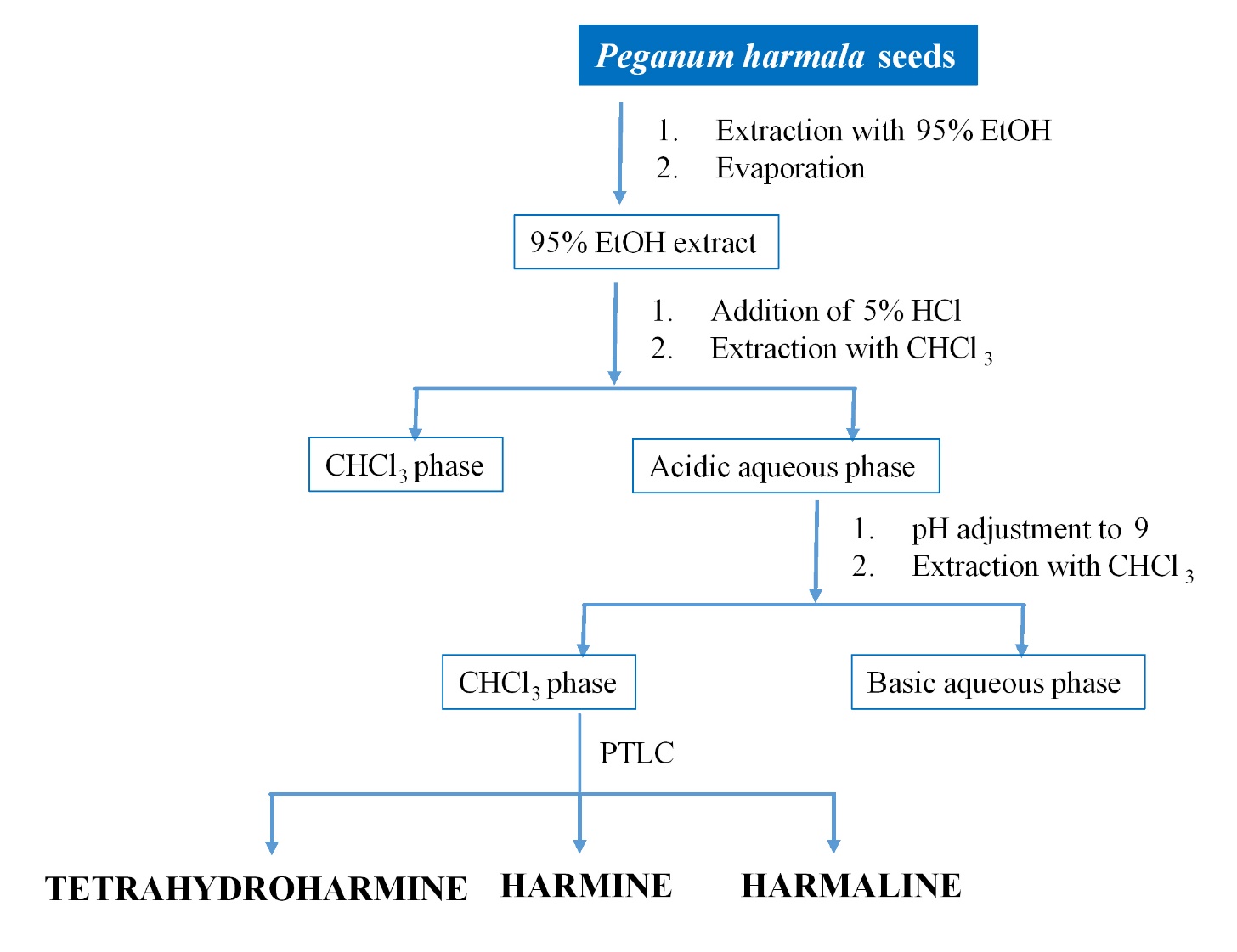


FIGURE S2. Scheme of isolation of alkaloids from *P. harmala* seeds. PTLC: preparative thin-layer chromatography


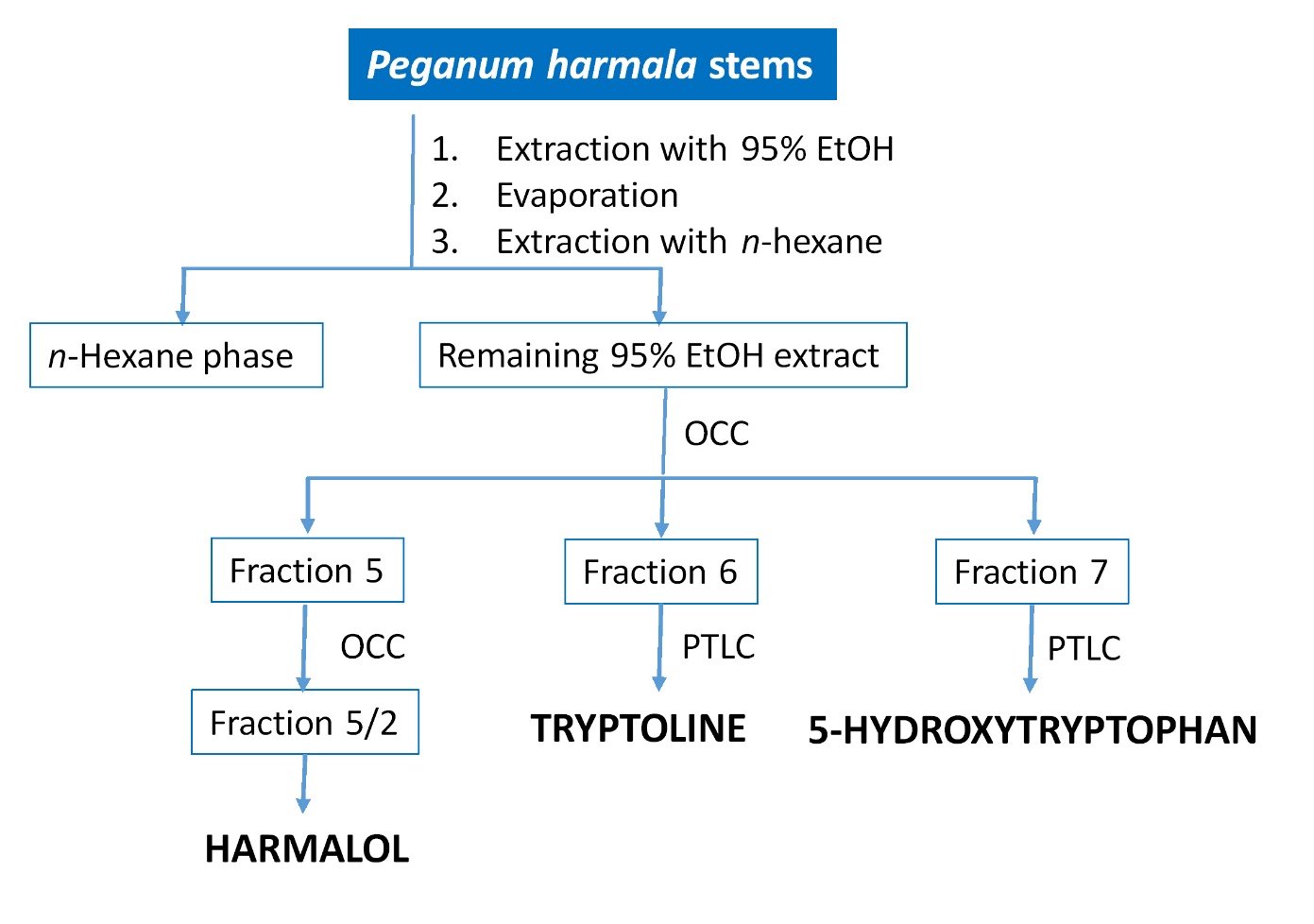


FIGURE S3. Scheme of isolation of alkaloids from *P. harmala* stem. OCC: open column chromatography; PTLC: preparative thin-layer chromatography


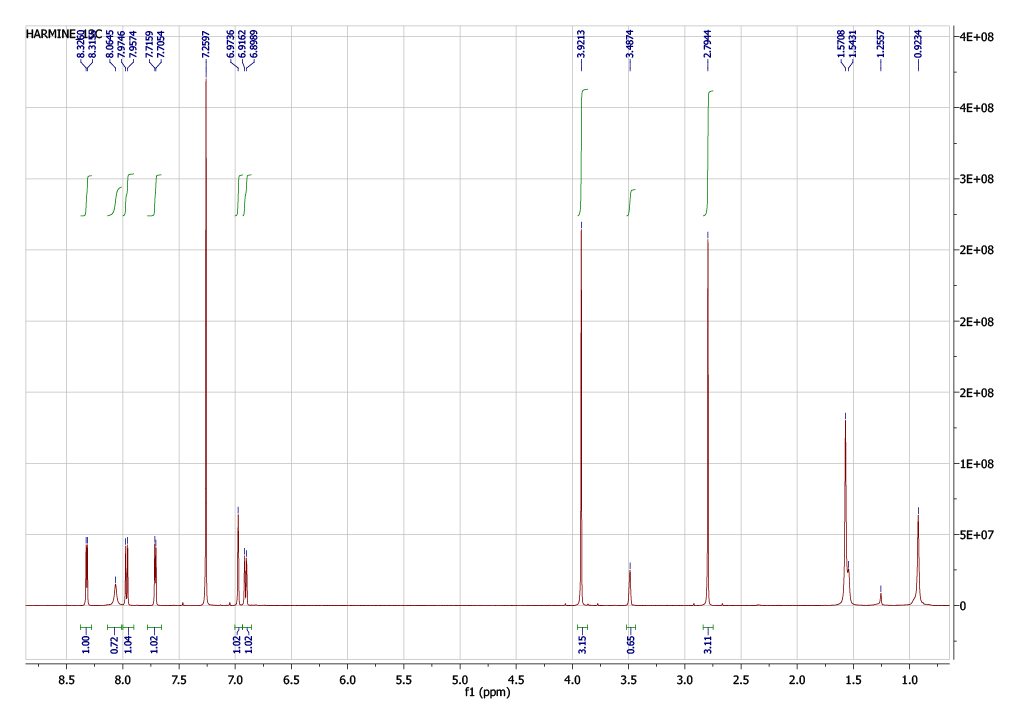


FIGURE S4. _1_H NMR properties of harmine


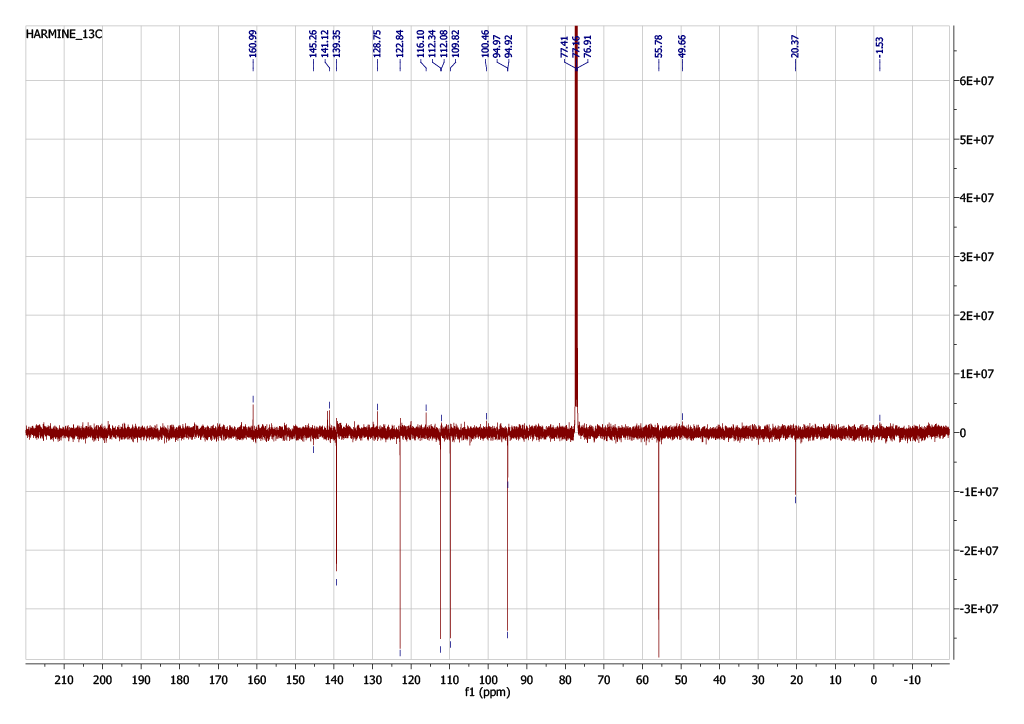


FIGURE S5. _13_C NMR properties of harmine


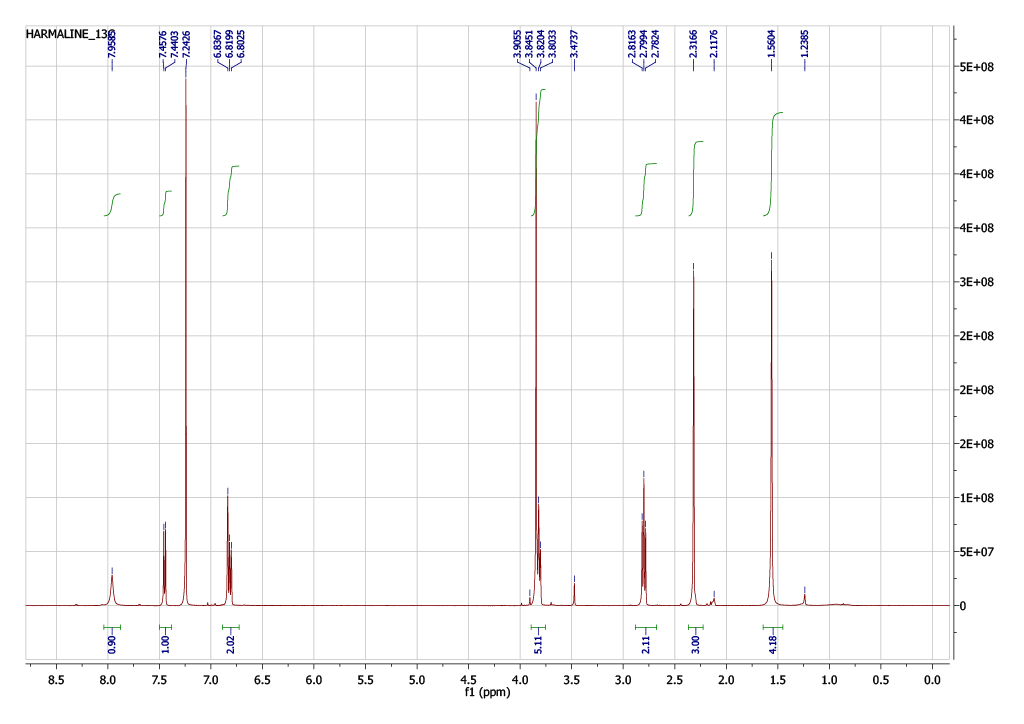


FIGURE S6. _1_H NMR properties of harmaline


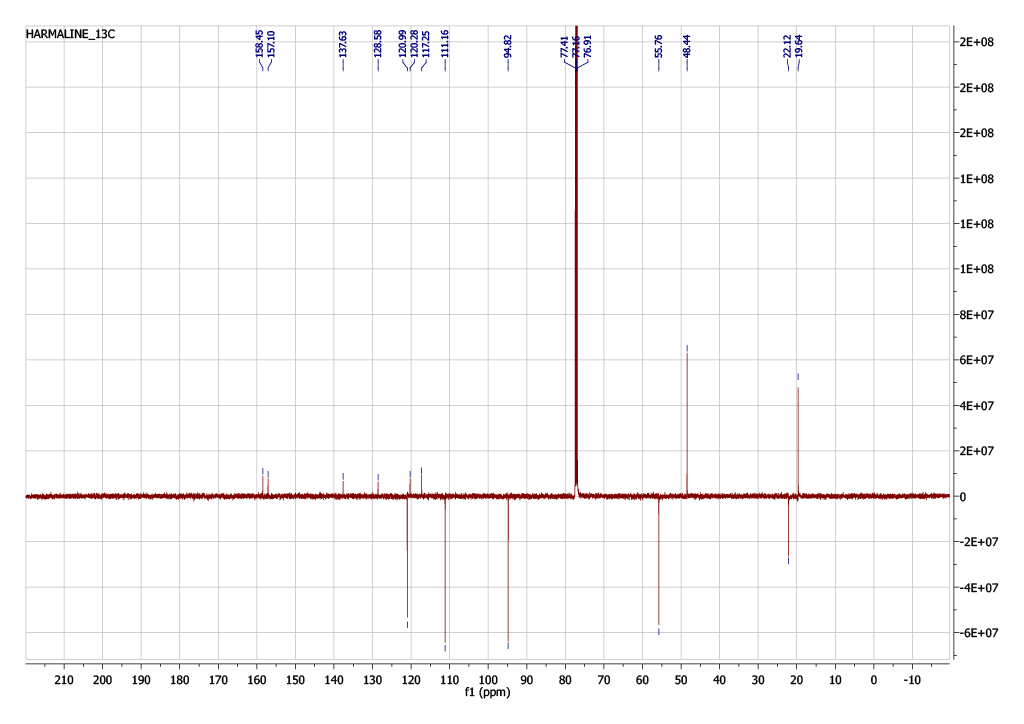


FIGURE S7. _13_C NMR properties of harmaline


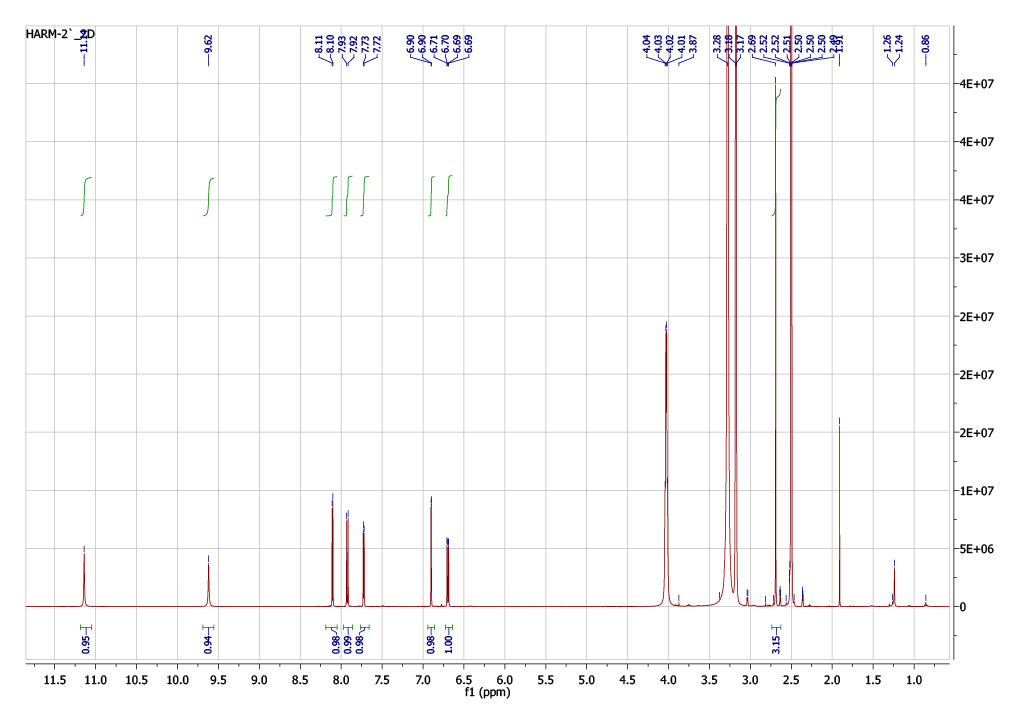


FIGURE S8. _1_H NMR properties of harmol


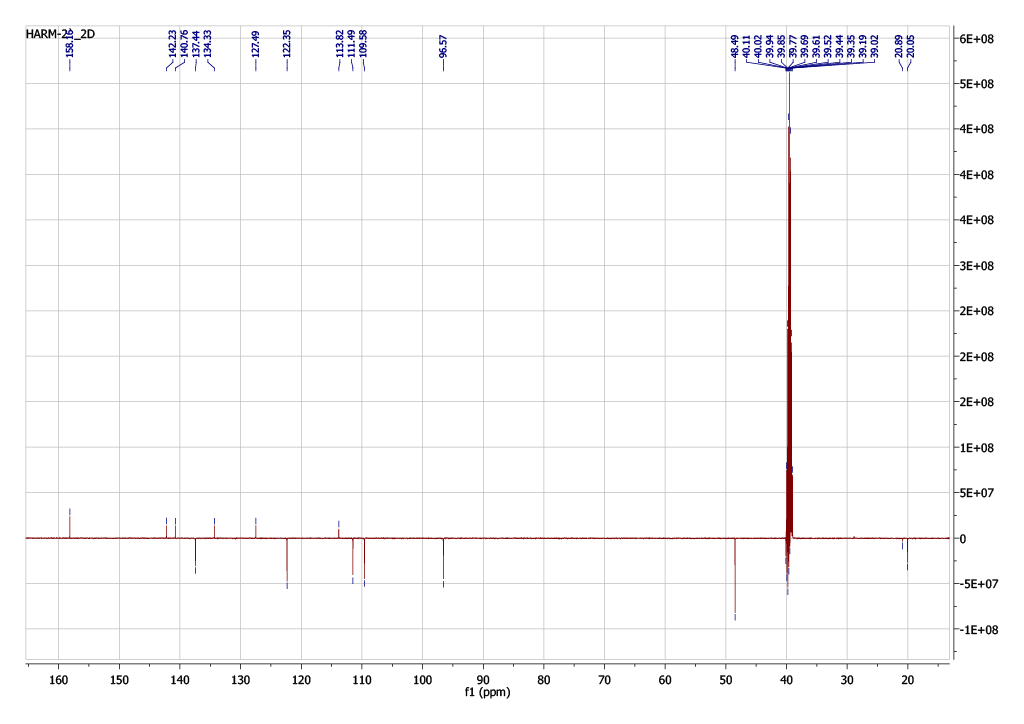


FIGURE S9. _13_C JMOD NMR properties of harmol

| a) |  | |  | |  |
| --- | --- | --- | --- | --- | --- |
| b) |  |  | |  | |
| c) |  |  | |  | |

FIGURE S10. HPLC chromatograms and UV spectra of *P. harmala* alkaloids at 340 nm and 254 nm: a) Harmine; b) Harmol; c) Harmaline

| Harmine | Harmol | Harmaline |
| --- | --- | --- |
|  |  |  |
| Y = aX + b  a = 3.53944e+009  b = 20813.2  R^2 = 0.9999397  R = 0.9999698 | Y = aX + b  a = 3.67608e+009  b = -118607  R^2 = 0.9986740  R = 0.9993368 | Y = aX + b  a = 3.04989e+011  b = 11416.9  R^2 = 0.9999765  R = 0.9999882 |

FIGURE S11. Calibration curves and properties of *P. harmala* alkaloids

Table S1. Antiproliferative activities of *P. harmala* extracts

| **Extract** | **Conc. (µg/ml)** | **Inhibition (%) ± SEM** | | | | | | | | |
| --- | --- | --- | --- | --- | --- | --- | --- | --- | --- | --- |
|  |  | **HeLa** | **SiHa** | **C33A** | **UPCI-SCC-131** | **UPCI-SCC-154** | **MCF-7** | **MDA-MB-231** | **A2780** | **NIH/3T3** |
| Seed | 20 | 39.29 ±1.19 | 32.32±1.31 | 47.17±1.85 | 23.25±2.69 | 38.43±1.22 | 22.55±2.50 | 29.51±2.24 | 15.13±2.29 | 10.66±1.53 |
|  | 60 | 52.67±2.30 | 57.26±1.23 | 64.03±1.92 | 88.19±0.74 | 81.64±1.14 | 59.37±2.11 | 49.78±1.71 | 70.67±2.04 | _* |
| Root | 20 | 67.33±0.67 | 61.82±0.61 | 68.40±0.72 | 83.09±1.08 | 68.44±1.83 | 76.37±1.57 | 69.16±1.60 | 91.88±1.18 | 17.47±3.55 |
|  | 60 | 96.61±0.36 | 96.90±0.99 | 97.34±0.21 | 96.78±0.43 | 88.06±1.56 | 94.30±1.16 | 96.97±0.48 | 98.65±0.21 | 59.49±1.64 |
| Stem | 20 | 26.97±1.32 | 33.18±1.96 | _ | 27.25±2.67 | 18.00±2.08 | 14.95±1.02 | 21.19±2.62 | _ | _ |
|  | 60 | 38.13±1.63 | 42.10±0.76 | 29.34±2.19 | 72.13±2.00 | 52.03±1.40 | 32.53±1.48 | 22.28±2.09 | _ | 15.89±2.17 |
| Capsule | 20 | 23.95±2.31 | 30.54±0.75 | _ | 35.02±3.31 | 24.23±1.12 | 11.74±1.70 | 17.52±2.93 | _ | _ |
|  | 60 | 35.95±1.59 | 40.43±1.43 | 28.34±1.80 | 74.13±2.53 | 55.45±0.58 | 25.31±2.25 | 20.42±2.65 | _ | 16.65±2.65 |
| Flower | 20 | 22.91±2.50 | 24.88±1.87 | _ | 30.38±3.13 | 25.38±1.42 | 11.05±2.32 | 16.68±2.96 | _ | _ |
|  | 60 | 33.14±1.90 | 38.90±1.20 | _ | 62.54±3.54 | 49.65±1.13 | 28.52±2.27 | 30.96±2.99 | _ | 18.34±1.78 |

*: The inhibition value is less than 10% and not numerically specified.

Table S2. Antiproliferative activities of *P. harmala* isolated alkaloids

| **Isolated compound** | **Conc. (µM)** | **Inhibition (%) ± SEM** | | | | | | | | |
| --- | --- | --- | --- | --- | --- | --- | --- | --- | --- | --- |
|  |  | **HeLa** | **SiHa** | **C33-A** | **UPCI-SCC-131** | **UPCI-SCC-154** | **MCF-7** | **MDA-MB-231** | **A2780** | **NIH/3T3** |
| Harmine | 10 | 28.38±2.07 | 54.27±1.816 | 45.27±1.57 | _* | 36.53±1.37 | 44.40±1.38 | 28.34±2.48 | 62.48±1.16 | 16.22±1.19 |
|  | 30 | 72.61±1.10 | 68.23±1.226 | 81.52±1.06 | 72.56±1.64 | 77.38±0.48 | 91.99±0.69 | 66.23±1.36 | 98.42±0.51 | 23.95±1.94 |
| Harmaline | 10 | 15.38±1.60 | 20.36±1.22 | _ | 29.76±3.37 | 21.42±1.11 | 17.58±1.32 | _ | _ | _ |
|  | 30 | 33.23±1.52 | 36.53±1.20 | _ | 61.68±4.60 | 43.19±2.03 | 30.50±1.92 | 18.48±1.49 | 16.76±2.90 | 14.80±1.80 |
| Harmol | 10 | 10.66±1.25 | 36.33±1.67 | 22.54±0.59 | 42.27±2.11 | 17.38±0.79 | 28.61±1.31 | 10.64±0.82 | 31.67±1.89 | 14.93±1.46 |
|  | 30 | 33.59±1.05 | 65.35±1.92 | 60.00±1.52 | 81.39±2.22 | 63.56±1.27 | 58.61±1.83 | 45.05±1.75 | 86.83±0.99 | 23.99±1.18 |
| Harmalol | 10 | _ | _ | _ | 10.22±1.60 | _ | _ | _ | _ | 10.82±1.62 |
|  | 30 | _ | 26.85±0.91 | _ | 52.99±3.09 | 25.53±1.57 | 36.53±1.64 | 10.99±1.26 | 13.39±2.88 | 25.87±3.43 |
| Tryptoline | 10 | _ | 19.05±1.23 | _ | 22.43±1.62 | 12.03±1.72 | 11.51±1.53 | _ | _ | 16.91±2.02 |
|  | 30 | 23.90±3.24 | 24.50±1.23 | _ | 43.73±3.97 | 33.12±3.26 | 26.79±2.22 | 14.75±1.31 | _ | 19.04±2.15 |
| Tetrahydro-harmine | 10 | 15.11 ±3.54 | 23.68±1.43 | _ | 34.24±3.11 | 16.24±3.09 | 20.93±1.31 | _ | _ | 14.53±1.80 |
|  | 30 | 29.90±2.15 | 27.54±0.94 | _ | 43.85±3.54 | 57.15±1.71 | 35.22±2.46 | 16.13±2.166 | 15.28±2.08 | 22.50±1.31 |
| 5-hydroxy tryptophan | 10 | _ | 14.16±1.16 | _ | 20.96±2.78 | 13.99±1.20 | 10.45±2.86 | _ | 13.55±2.80 | _ |
|  | 30 | 13.80±2.09 | 19.58±1.86 | _ | 43.80±2.95 | 30.64±2.91 | 30.08±1.44 | 10.54±1.66 | 20.15±2.07 | _ |
| Cisplatin | 10 | 42.61 ±2.33 | 88.64 ± 0.50 | 85.98 ±1.05 | 95.63 ± 1.49 | 87.40 ± 1.72 | 66.91 ± 1.81 | _ | 83.6 ± 1.20 | 76.74 ± 1.26 |
|  | 30 | 99.93 ±0.26 | 90.18 ± 1.78 | 98.66 ±0.21 | 95.09 ± 1.57 | 92.72 ± 1.67 | 96.80 ± 0.35 | 71.47 ± 1.20 | 95.0 ± 0.30 | 96.90 ± 0.25 |

*: The inhibition value is less than 10% and not numerically specified

FIGURE S12. Representative dose–response curves of antiproliferative activity for *P. harmala* root
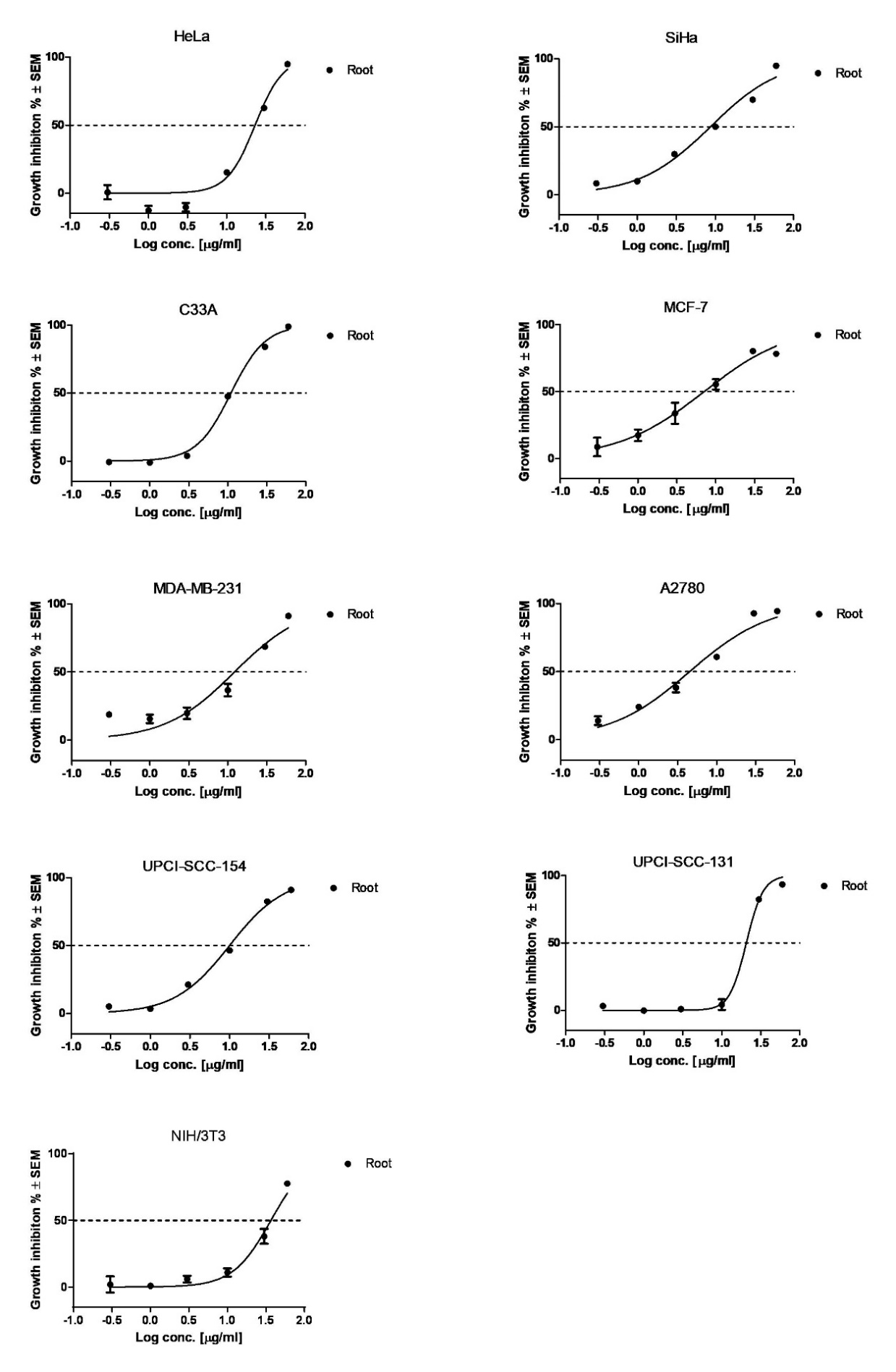
extract


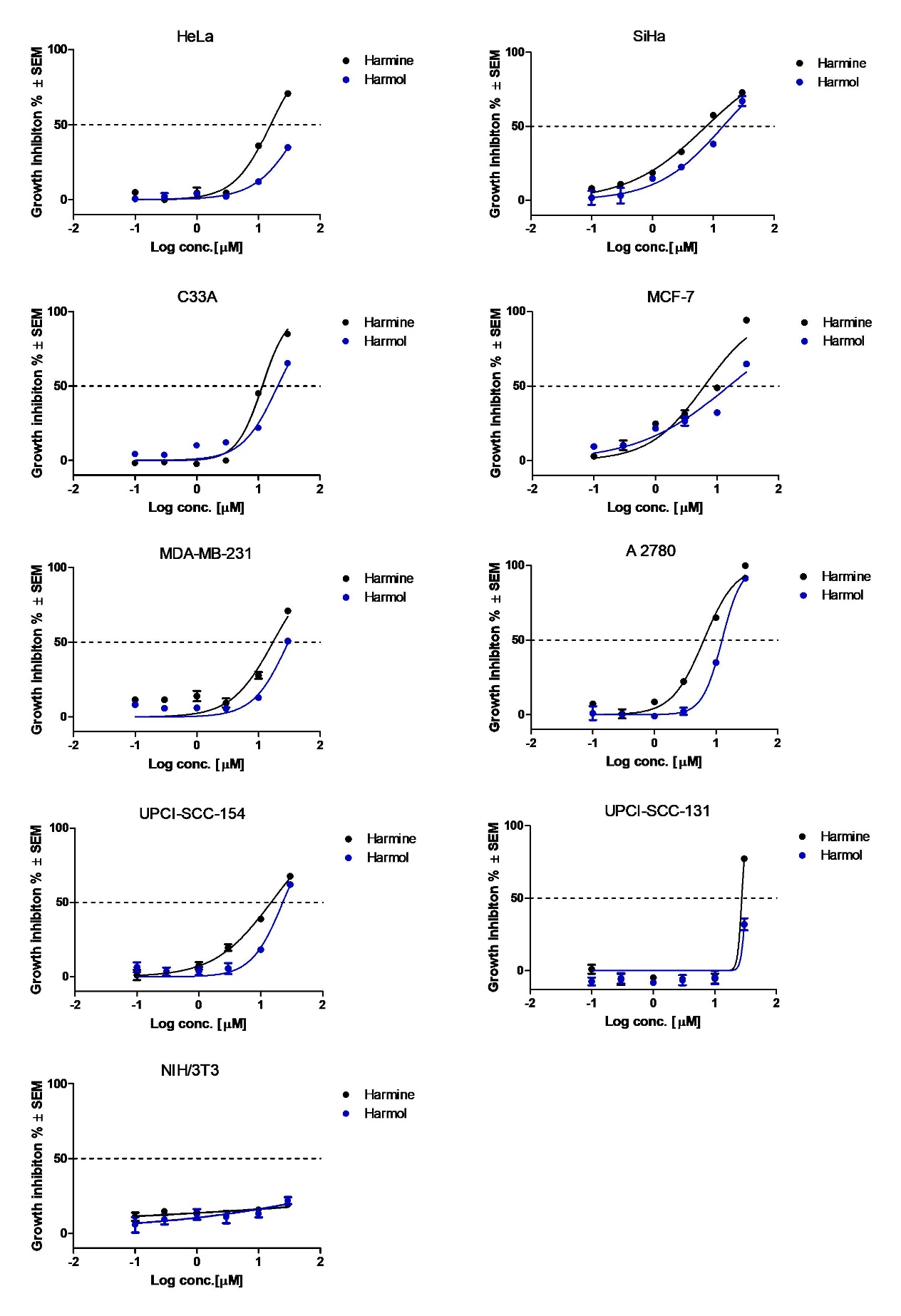
FIGURE S13. Representative dose–response curves of antiproliferative activity for harmine and harmol


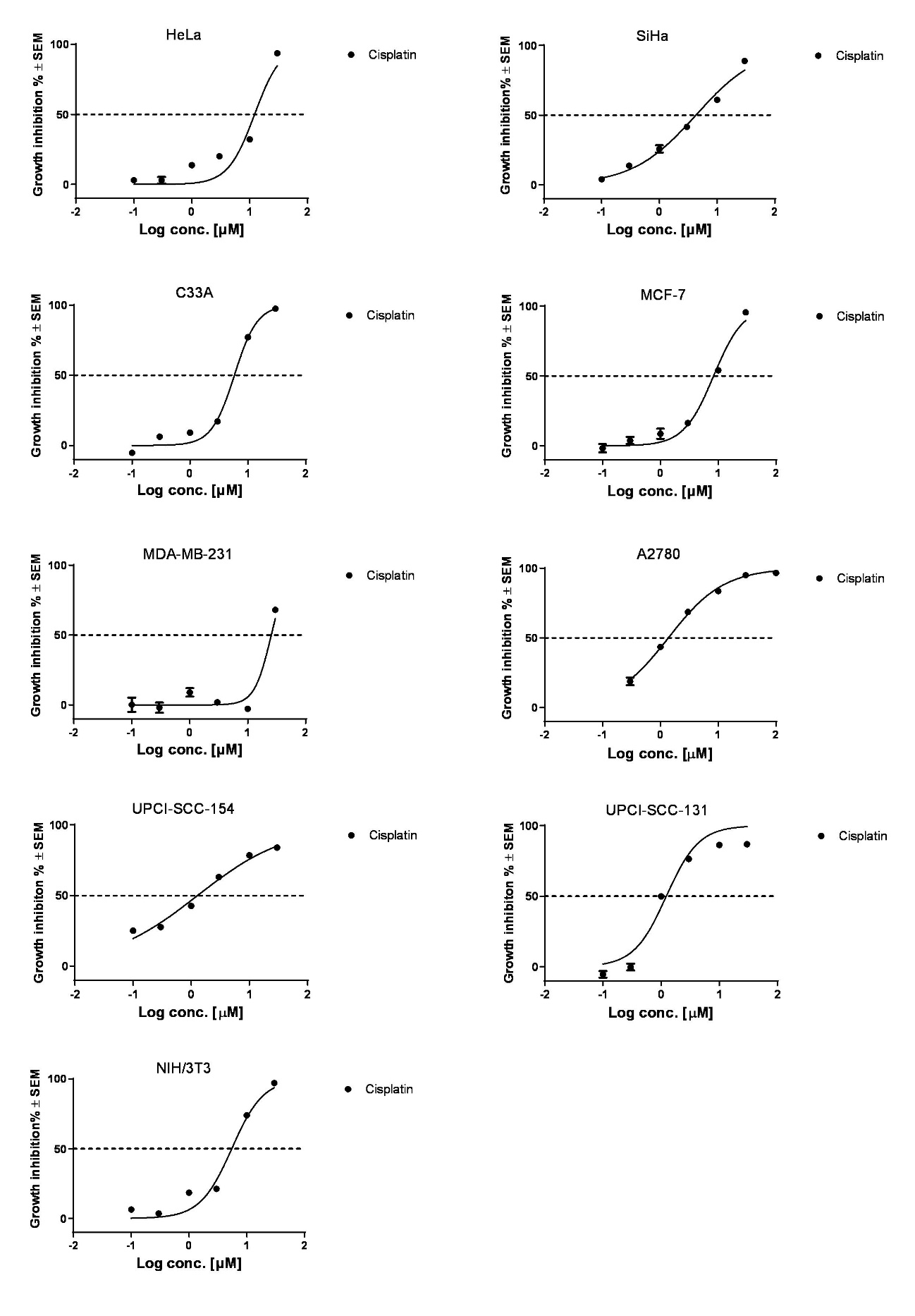
FIGURE S14. Representative dose–response curves of antiproliferative activity for cisplatin.

Some curves shown in the figure are based on data reported in our earlier publication (https://doi.org/10.3390/pharmaceutics17101253)


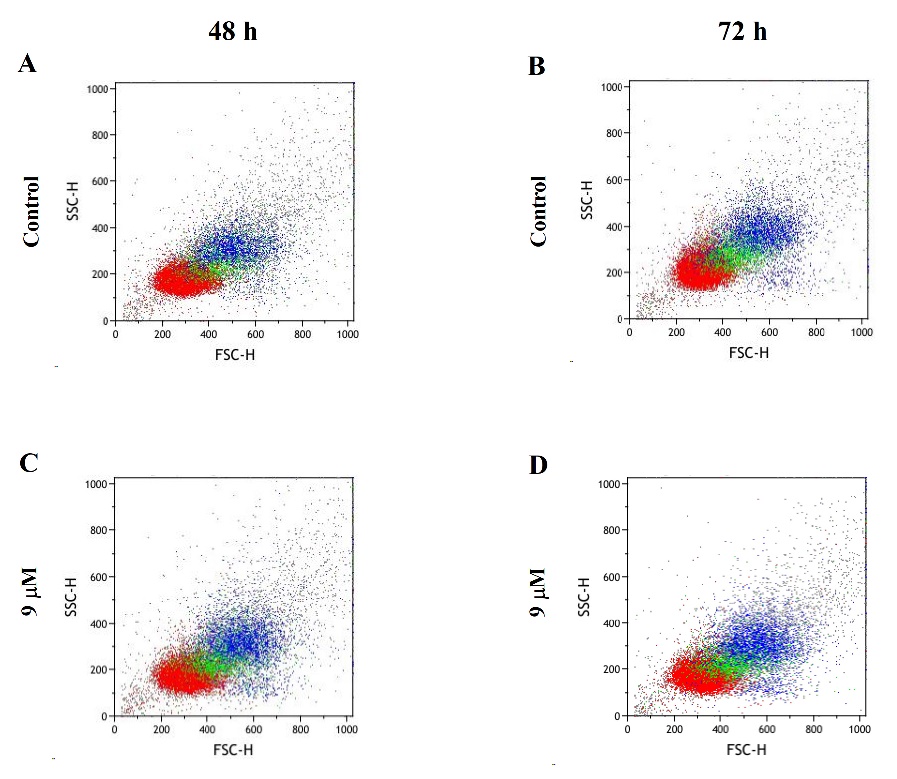


FIGURE S15. Harmine induces cell cycle arrest in the SiHa cells. Representative dot plots: Controls **(A, B)** 48 h and 72 h, respectively, and treated cells **(C, D)** 48 h and 72 h post-treatment, respectively. Colors indicate cell cycle phases: yellow (Sub-G1), red (G1), green (S), and blue (G2/M).


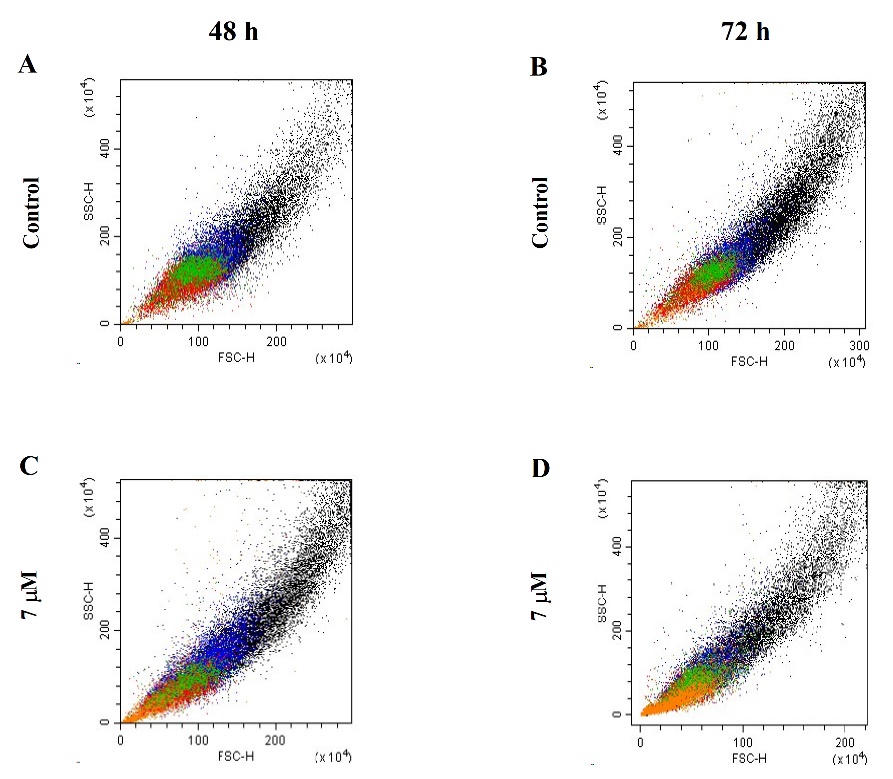


FIGURE S16. Harmine induces cell cycle arrest in the UPCI-SCC-154 cells. Representative dot plots: Controls **(A, B)** 48 h and 72 h, respectively, and treated cells **(C, D)** 48 h and 72 h post-treatment, respectively. Colors indicate cell cycle phases: yellow (Sub-G1), red (G1), green (S), and blue (G2/M).
